# Sexual and gender minority health in the Middle East and North Africa Region: A scoping review

**DOI:** 10.1016/j.ijnsa.2022.100085

**Published:** 2022-06-27

**Authors:** Sarah Abboud, Cindy Veldhuis, Suha Ballout, Fatima Nadeem, Kate Nyhan, Tonda Hughes

**Affiliations:** a University of Illinois Chicago, College of Nursing, Chicago, IL, United States of America; b Columbia University, School of Nursing, New York, New York, United States of America; c University of Massachusetts Boston, College of Nursing and Health Sciences, Boston, Massachusetts, United States of America; d University of Windsor, Ontario, Abbreviation:; e Yale University Cushing/Whitney Medical Library, New Haven, Connecticut, United States of America

**Keywords:** Middle East and North Africa, Lesbian, Gay, Bisexual, Transgender, Queer, Sexual and gender minority, Health, Scoping review

## Abstract

**Background:**

Researchers in studies from multiple countries suggest that sexual and gender minority people experience high rates of violence, stigma, and discrimination, as well as mistrust of health care providers and systems. Despite growing evidence related to sexual and gender minority health in North America and Europe, we know little about the health of this population in the Middle East and North Africa.

**Objectives:**

We aimed to comprehensively examine the literature related to the health of sexual and gender minority people in the Middle East and North Africa and to identify research gaps and priorities.

**Design:**

We conducted a scoping review informed by the framework recommended by Arksey and O'Malley and the Preferred Reporting Items for Systematic Reviews and Meta-Analyses extension for Scoping Reviews (PRISMA-ScR) tool.

**Data sources:**

We searched the following databases: PubMed (using Medline All on the Ovid platform), PsycINFO (Ovid), CINAHL (Ebsco), and Embase (Ovid). The search strategy combined terms for the geographic region of interest (Middle East and North Africa) and the population of interest (sexual and gender minority). Each was operationalized using multiple search terms and, where available, controlled vocabulary terms.

**Review Methods:**

Research articles were identified and assessed for inclusion using an explicit strategy. Relevant information was extracted and synthesized to present a descriptive summary of existing evidence.

**Results:**

Research designs of the 98 articles we reviewed included quantitative (*n* = 73), qualitative (*n* = 20), and mixed methods (*n* = 5). Most studies were conducted in Lebanon (*n* = 33), Pakistan (*n* = 32), and Iran (*n* = 23) and focused mainly on gender minority individuals (*n* = 46) and men who have sex with men (*n* = 32). Five themes emerged from the review: sexual health (52; 53%); mental health (20; 20%); gender identity (17; 17%); violence and discrimination (7; 7%); and experiences with the healthcare system (2; 2%). Although researchers focused on multiple health outcomes in some studies, we included them under the theme most closely aligned with the main objective of the study.

**Conclusion:**

Although our study is limited to few countries in the Middle East and North Africa region, we found that sexual and gender minority individuals face multiple adverse sexual and mental health outcomes and experience high rates of stigma, discrimination, and violence. More research is needed from countries outside of Lebanon, Pakistan, and Iran, including community-based participatory approaches and multi-level intervention development. Nurses and other healthcare providers in the region need training in providing inclusive care for this population.

## 1 Background

Sexual and gender minority, also known as lesbian, gay, bisexual, transgender, and queer people, experience high rates of violence, stigma, and discrimination (Rothman et al., 2011; Sadika et al., 2020; Williams et al., 2021), lack of cultural competence in health care settings (Alencar Albuquerque et al., 2016; Lerner and Robles, 2017; Lisy et al., 2018), and high rates of health disparities (Williams et al., 2021; Zeeman et al., 2019). These health disparities are attributable to multiple minority stressors (Meyer, 2003) that function at the structural (anti-sexual and gender minority policies and laws), interpersonal (victimization, discrimination), and individual (internalized stigma, identity concealment) levels and contribute to increased stress and health burdens (Hafeez et al., 2017; Hatzenbuehler, 2016; Lamontagne et al., 2018; Pachankis et al., 2017; Pachankis et al., 2020). Previous researchers have found higher rates of depression, anxiety, suicidal ideation, alcohol and drug use, human immunodeficiency virus (HIV) risk behaviors, and unhealthy coping mechanisms among sexual and gender minority people compared to heterosexual and cisgender people (Flentje et al., 2020; Hatzenbuehler and Pachankis, 2016; Richman and Hatzenbuehler, 2014). A cisgender individual is defined as someone whose gender identity is congruent with their sex assigned at birth. For example, sexual minority men are twice as likely as heterosexual men to report a lifetime history of depression and anxiety (Cochran and Mays, 2009; Meyer, 2003). Despite growing evidence related to these health disparities in North America and Europe, we know little about the health of sexual and gender minority people living in the Middle East and North Africa.

Many countries in the Middle East and North Africa have laws that criminalize same-sex behaviors or relationships. For example, Article 534 of the Lebanese Criminal Code states that "any sexual intercourse contrary to the order of nature" is punishable with up to a year in prison (Al Farchichi and Saghiyeh, 2021). In Iran, same-sex relations are illegal, but the country allows transgender individuals the right to have their identity lawfully recognized. However, this recognition is conditional based on a psychiatric diagnosis of gender identity disorder and the completion of gender affirmation surgery (which is subsidized by the government) (Being Transgender in Iran 2016). These laws create structural stigma, which is defined as “societal-level conditions, cultural norms, and institutional practices that constrain the opportunities, resources, and wellbeing for stigmatized populations” (Hatzenbuehler and Link, 2014p. 2) and compound the complex forms of minority stress at the intrapersonal and interpersonal levels that sexual and gender minority people face. Stigma and discrimination have been identified as contributing to the persistent health disparities among sexual and gender minority people (Human Rights Watch 2018).

Social, religious, and political contexts in some parts of the Middle East and North Africa have shifted over the past decade towards intolerant forms of conservative extremism that have set back the work of community activists towards sexual and gender minority rights (Human Rights Watch 2018; Masri, 2020). At the same time, we have witnessed a rise in advocacy for sexual and gender minority rights from grassroot activists, community organizers, and professional groups (Human Rights Watch 2018; Masri, 2020). Despite a parallel increase in research, many gaps in knowledge remain related to sexual and gender minority health.

As the largest healthcare profession, nurses should play a significant role in reducing health disparities among vulnerable populations such as sexual and gender minority people. With the significant impact of social determinants on health and wellbeing, nurses should be adequately trained in providing safe and competent care (World Health Organization 2019) and in mitigating factors that threaten the health and safety of sexual and gender minority people. However, no studies have been conducted in the Middle East and North Africa examining the role of nurses in providing care to sexual and gender minority people; in one study, researchers reported general mistrust toward healthcare providers and that they lack the appropriate training and education to provide competent care (Abboud et al., 2020).

The context in which sexual and gender minority people live in the Middle East and North Africa creates unique health disparities in this population that remain poorly understood. Therefore, the overall objective of our scoping review was to comprehensively examine the literature related to the health of sexual and gender minority people in the Middle East and North Africa and to identify research gaps and priorities. Implications for nurses are also discussed.

## 2 Methods

### 2.1 Design

Scoping reviews are appropriate when there is minimal literature on a topic, when methodological approaches are heterogeneous, and when the research question/objectives are broad in nature Arksey and O'Malley (2005). In line with the purpose of a scoping review Arksey and O'Malley (2005), we aimed to explore and map the literature for important concepts and present an overview of a potentially large and diverse number of research studies pertaining to sexual and gender minority health in the Middle East and North Africa. We used the methodological framework for scoping reviews recommended by Arksey and O'Malley (2005). The framework includes five steps: 1) identify the research question; 2) identify relevant studies; 3) select the studies; 4) extract and chart the data from the relevant studies; and 5) synthesize and report the results. We did not assess the risk of bias or the quality of the studies because, unlike systematic reviews, the aim of scoping reviews is to provide a descriptive overview of the reviewed studies and identity gaps and future research priorities (Arksey and O'Malley, 2005; Tricco et al., 2018).

Our research team included five cisgender individuals who live in the United States (US) and one in Canada whose educational backgrounds are in nursing, psychology, public health, global health, and library and information science. Half of the team identifies as lesbian, gay, or bisexual. Among those living in the US, two were born and raised in Lebanon and are closely involved in sexual and gender minority research and advocacy in Lebanon and the region. We acknowledge that our multiple identities and experiences both in Lebanon and globally inform and influence our research interests, including the aims and approach of this study. To address our biases, we met regularly, debriefed, and discussed the inclusion/exclusion criteria, data extraction and synthesis, and presentation of findings and implications to ensure the rigor of the study.

### 2.2 Data sources and search strategy

We searched the following databases: PubMed (through Medline All on the Ovid platform), PsycINFO (Ovid), CINAHL (Ebsco), and Embase (Ovid). The search strategy combined terms for the geographic region of interest (Middle East and North Africa) and the population of interest (sexual and gender minority). Each was operationalized with multiple search terms and, where available, controlled vocabulary terms. In Scopus, which indexes materials from fields other than biomedicine, the search strategy included a filter based on journal subjects. Search terms for the Middle East and North Africa included the combination of names of all countries in this region and their capitals, as defined by the World Health Organization (WHO EMRO | Countries 2019) and the World Bank (Middle East and North Africa 2019). Search terms for sexual and gender minority people included various phrases used in health and social sciences research. We used and expanded upon the sexual and gender minority search terms from a recent scoping review on substance use among sexual and gender minority youth (Kidd et al., 2018) and the search terms recommended in a systematic review of sexual and gender minority search terms (Lee et al., 2016). Our search strategy was guided by the research team and designed and conducted by the fifth author, who is an experienced health information specialist. The Medline search strategy was peer reviewed by an independent information specialist using the PRESS checklist. Supplement 1 provides the search terms and syntax used.

### 2.3 Criteria and process

We used the following inclusion criteria: 1) studies conducted in the Middle East and North Africa based on country definitions from both the World Health Organization and the World Bank: Afghanistan, Algeria, Bahrain, Djibouti, Egypt, Iran, Iraq, Jordan, Kuwait, Lebanon, Libya, Mauritania, Morocco, Oman, Palestine, Pakistan, Qatar, Saudi Arabia, Somalia, Sudan, Syria, Tunisia, United Arab Emirates, or Yemen; 2) studies conducted in Israel that included Arabs/Palestinians; 3) peer-reviewed studies focused on sexual and gender minority health outcomes; 4) quantitative, qualitative, or mixed-methods designs (interventional or observational); and 5) studies written in English, Arabic, or French. Consistent with the World Health Organization's definition of health, we included any empirical paper that described a health outcome related to physical, mental, or social well-being (World Health Organization(WHO) 2019). We excluded: 1) gray literature (e.g., conference presentations, theses/dissertations, reports, commentaries or editorials, and books); 2) systematic/literature reviews; 3) articles that reported on psychometric development and testing; 4) articles that reported on healthcare professionals’ or the public's attitudes of sexual and gender minority people; 5) articles that reported on population estimates of sexual and gender minority groups that did not include health outcomes; 6) studies conducted with Middle East and North Africa migrants or refugees in a non-Middle East and North Africa country; 7) studies published before the year 2000; and 8) studies conducted in Israel that did not include separate data for Arabs/Palestinians (based on the definitions of the Middle East and North Africa by the World Health Organization and the World Bank that exclude Israel and given the sociocultural differences between Arabs/Palestinians and Israelis residing in Israel).

Duplicate articles were removed after the final search was completed in March 2021, and all citations were exported to Covidence, a systematic review manager. Two independent raters, the first and fourth authors, evaluated the eligibility of all identified titles and abstracts for inclusion using Covidence. Disagreements were resolved by discussion and consensus. Full-text articles were then independently evaluated for inclusion by the same raters using the same inclusion and exclusion criteria.

### 2.4 Data extraction and synthesis

The following data were extracted by the first and fourth authors to summarize key features of the studies: author(s) and year of publication, country, source of funding, study objectives, methods, sample characteristics, and main findings/themes. The studies were initially grouped based on country and then regrouped based on themes of common health outcomes. The research team met regularly to discuss and organize the studies based on their main objectives to fall under the five common themes: sexual health outcomes, mental health outcomes, violence and discrimination, experiences with the healthcare system, and gender identity. We also paid special attention to methodological trends, the range and scope of the findings, and the gaps in the literature.

## 3 Results

**Fig. 1:**
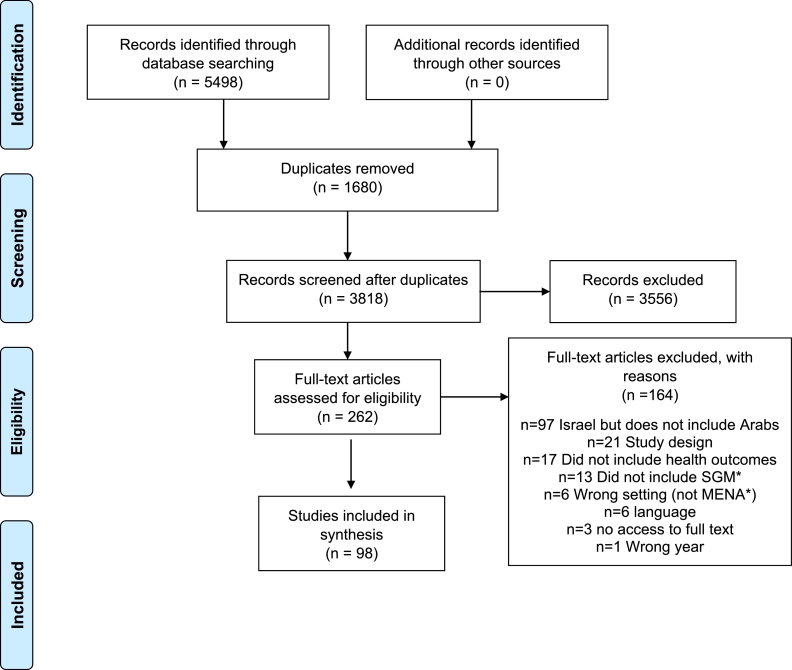
PRISMA flow diagram for scoping review showing literature search and selection. SGM: sexual and gender minority; MENA: Middle East and North Africa.

We identified a total of 5498 articles through electronic database searches conducted in April 2020 and updated in March 2021. Following removal of duplicates (1680), 3818 article titles and abstracts were screened; 3556 were excluded as not relevant, leaving 262 full-text articles to be screened. After reading the full texts, 162 articles were excluded for one or more of the following reasons: studies conducted in Israel but did not include Arab/Palestinian participants (*n* = 97); study designs such as sexual and gender minority population estimates and did not include health outcomes (*n* = 21); not health-related (*n* = 17); study population did not include sexual and gender minority people (*n* = 13); conducted with Middle East and North Africa migrants/refugees but not conducted in the Middle East and North Africa region (*n* = 6); language not spoken by any of the co-authors (*n* = 6); no access to full text (*n* = 3); and published before the year 2000 (*n* = 1). Articles that were excluded because of language (five Farsi and one Hebrew) or because of no access to full text are listed in Supplement 2. After these exclusions, 98 articles were included for review (Fig. 1).

### 3.1 Characteristics of studies

**Fig. 2:**
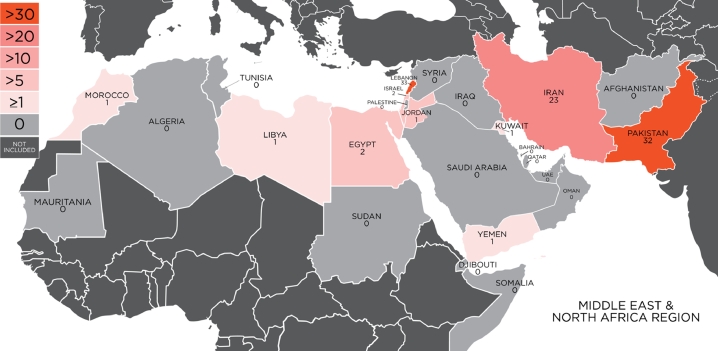
Heat map of number of research articles in each country in the MENA region.

Research designs of the studies were quantitative (*n* = 73), qualitative (*n* = 20), and mixed-methods (*n* = 5). As shown in Fig. 2, most studies were conducted in Lebanon (*n* = 33), Pakistan (*n* = 32), and Iran (*n* = 23), with the majority focused on gender minority people (transgender, transsexual, hijra [term used in Pakistan to describe gender minority individuals]; *n* = 58), or sexual minority men (*n* = 51). Several studies included both sexual and gender minority participants. With the exception of one article that was published in French, all were in English. Sample sizes ranged from three to 50 participants for the qualitative studies and from 10 to 43,522 participants for the mixed-methods and quantitative studies. Additional study characteristics are summarized in Table 1.

**Table 1:** Study characteristics (*N* = 98).

Characteristics	N
**Publication year**	
2000–2010	11
2011–2016	43
2017–2021	44
**Location**	
Lebanon	33
Pakistan	32
Iran	23
Egypt	2
Israel	2
Jordan	1
Kuwait	1
Libya	1
Morocco	1
Yemen	1
Multiple MENA countries	1
**Study design**	
Quantitative (*n* = 73):	
Cross-sectional	64
Experimental	4
Descriptive comparative	2
Secondary data analysis	1
Case control	1
Case study	1
Qualitative (including one secondary qualitative data analysis)	20
Mixed methods	5
**Sample**	
Transgender, transsexual, hijra (term used in Pakistan to describe gender minority individuals)	46
Men who have sex with men/gay/bisexual cisgender men	32
Male sex workers, female sex workers, and hijras	10
Lesbian, gay, bisexual cisgender men and women	4
Men who have sex with men /female sex workers	3
Lesbian, bisexual cisgender women	1
Men who have sex with men and transgender	1
Lesbian, gay, bisexual, transgender	1
**Sampling**	
Convenience and/or purposive	39
Respondent driven sampling and/or long chain peer referral	23
Snowball	11
Peer recruitment/personal network/network sampling	6
Stratified random selection	5
Multiple sampling methods	14
**Source of funding**	
Not disclosed	42
Foundations	29
National Institutes of Health (United States)	15
Not funded	5
University	6
Foundation and National Institutes of Health (United States)	1

### 3.2 Themes

Five themes emerged from the review: sexual health (*n* = 52; 53%), mental health (*n* = 20; 20%), violence and discrimination (*n* = 7; 7%), experiences with the healthcare system (*n* = 2; 2%), and studies about gender (*n* = 17; 17%). Although in some studies, researchers assessed multiple health outcomes, we categorized them in the theme corresponding to the closest fit with their main research objective. In the following section, we report findings of the studies organized by themes. Despite the use of multiple and sometimes imprecise terminology by the authors of the studies reviewed, we chose to use the terms “sexual minority” and “gender minority” to consistently refer to the diverse samples in the studies. More detailed information about the composition of samples is found in the relevant table for each section.

### 3.3 Sexual health

In more than half of the studies (*n* = 52), researchers focused on sexual health, specifically rates, testing, and risks for HIV or other sexually transmitted infections (Table 2). These studies were primarily conducted in Lebanon (*n* = 23) or Pakistan (*n* = 17); three were conducted in Iran, two in Egypt, two in Israel, and one each in Jordan, Libya, Morocco, and Yemen. One study included participants from multiple Middle East and North Africa countries. We first describe the studies conducted with sexual minority people (*n* = 39), followed by the studies conducted with gender minority people (*n* = 21). Eight studies included both sexual and gender minority participants.

**Table 2:** Sexual health (*n* = 52).

**First Author (Year); Country**	Study aims	Study design and sample characteristics	Relevant findings
1 El-Sayyed (2008); Egypt	Assess risk behaviors for HIV/AIDs among men who sex with men in Cairo, Egypt.	**Design**: Quantitative cross-sectional**Age**: Range: 15–47 years (majority of participants being 15–25 years). **Gender:** Cisgender male **Sexual orientation:** Men who sex with men**Sample size:***N* **=** 73	Knowledge about HIV/AIDS and condom use, and a history of sexually transmitted infections were positively associated with educational level. HIV screening: one participant (1.4%) tested positive. Condoms were never used by 52.1% of participants. Condom use increased with age and educational level. Men who sex with men engaged in penetrative and receptive anal sex (65.8%) and with more than 1 partner (76.7%). More than half had more than 3 partners per week (53.4%).
2 Elmahy (2018); Egypt	Develop a framework for using the internet to survey gay Egyptians; and to assess risky sexual behaviors and preventive health behaviors.	**Design**: Quantitative cross-sectional**Age:** Mean = 27 **Gender:** Cisgender male **Sexual orientation:** Homosexual and bisexual**Sample size:***N* = 461	Condom use was consistent in 17% of participants and was significantly associated with practicing religion. 34% of participants have tested for HIV; 3% had a previous history of sexually transmitted infections. HIV testing was associated with hepatitis B vaccination and other sexually transmitted infections testing. HIV testing was lower among student and non-working individuals compared to working individuals.
3 Zamani (2010); Iran	Report the proportion of injecting drug users who have had sex with other men and the prevalence of and risk characteristics related to HIV-1 infection.	**Design:** Quantitative cross-sectional **Age**: Mean = 26 **Gender:** Cisgender male **Sexual orientation:** Men who sex with men and non-men who sex with men **Sample size:***N* = 307	Men who sex with men injecting drug users had a higher history of exchanging money or drugs for sex than other injecting drug users. Rate of condom use during their last sexual encounter was low and comparable among men who sex with men injecting drug users and non- men who sex with men injecting drug users participants (24% vs. 19%, respectively). In both groups of injecting drug users, slightly over 40% of the participants reported having had at least one prior HIV test. Based on our HIV testing, there was no significant difference in the prevalence of HIV-1 infection between men who sex with men injecting drug users and non- men who sex with men injecting drug users (22% vs. 18.5%, respectively)
4 Moayedi-Nia (2019); Iran	Investigate the prevalence of HIV and related high-risk behaviors among transgender women in Iran.	**Design:** Quantitative cross-sectional **Age:** Mean = 28 **Gender:** Transgender women **Sexual orientation:** Not specified **Sample size:***N* = 104	Two participants were diagnosed with HIV (1.9%). Receptive anal intercourse was the most common sexual practice (65.8%). The prevalence of condom use for always or most of the time was 39.7%. The lack of condom use was due to either trusting non-paying partners or opposition from casual and paying partners.
5 Eftekhar (2020); Iran	Identify the factors that underlie the higher prevalence of high-risk sexual behaviors in transgender women	**Design:** Qualitative **Age**: Range: 21–34 years **Gender:** Transgender women **Sexual orientation:** Not specified **Sample size:***N* = 15	Themes identified: The role of intimate relationships in sexual identity development; Engaging in higher risk behaviors during intimate relationships
6 Mor (2015); Israel	Assess the knowledge of Arab men who sex with men regarding HIV transmission, attitudes towards condom use and sexual practices compared with Jewish men who sex with men	**Design:** Quantitative cross-sectional**Age:** 18 years or older **Gender:** Cisgender male **Sexual orientation:** Men who sex with men**Sample size:***N* = 2117	Arab men who sex with men knowledge regarding HIV transmission was generally lower and their attitudes towards condoms and safe sex were less favorable than those of Jewish men who sex with men. HIV testing and previous HIV/sexually transmitted infections diagnoses were reported less commonly among Arab men who sex with men than Jewish men who sex with men. Arab men who sex with men had their first sexual encounter at an earlier age, had a greater number of sexual partners, were less likely to perform receptive anal intercourse, were more likely to pay for sex and perform unprotected anal intercourse, and were less likely to engage in group sex than Jewish men who sex with men.
7 Zuckerman (2019); Israel	Retrospectively assess the spread of HIV-1 among the Arab men who sex with men and Jewish men who sex with men diagnosed in Israel between 2005 – 2016.	**Design**: Quantitative cross-sectional **Age**: Mean = 33 **Gender**: Cisgender male **Sexual orientation:** Men who sex with men **Sample size:***N* = 1143 participants	While 1070 Jewish men who sex with men were diagnosed with HIV-1 between 2005 and 2016, only 6.4% were Arab men who sex with men. The prevalence of the various HIV-1 subtypes infecting Arab men who sex with men and Jewish men who sex with men was nearly identical, suggesting shared transmission networks between the two populations.
8 Alkaiyat (2014); Jordan	Identify sociocultural determinants of condom use and HIV testing among men who have sex with men in Jordan.	**Design:** Cross-sectional mixed methods study**Age:** Older than 17 years of age **Gender:** Cisgender male **Sexual orientation:** Homosexual**Sample Size:***N* = 97	Condom use: 10% reported always using condoms. HIV testing: Of those who had ever tested, 92% had done so voluntarily. ‘‘No need for the test’’ was the most frequent reason for not testing, reported by 52%. Stigma was reported by 51% as an obstacle to testing. Qualitative data showed that stigma mainly arose from interactions with healthcare workers, compounded by concerns about the confidentiality of testing. HIV knowledge: All respondents had heard about HIV/AIDS; 32% believed that treatment is available for HIV/AIDS, but only 12% mentioned antiretroviral treatment.
9 Mahfoud (2010); Lebanon	Measure HIV prevalence and associated risk factors among female sex workers, injecting drug users (injecting drug users) and men who have sex with men in Lebanon and the prevalence of hepatitis B virus and hepatitis C virus among injecting drug users.	**Design:** Quantitative cross-sectional **Age:** 16 years or older **Gender:** Cisgender female and male **Sexual orientation:** Not specified **Sample size:***N* = 317	3.7% of men who sex with men were positive for HIV; 22% of men who sex with men had a history of HIV testing; 67% of men who sex with men reported feeling at risk for HIV. 52% reported first anal intercourse when under 18 years of age. 63% of men who sex with men used a condom every time they had sex with a regular non-commercial partner, but only 39% did so every time they had sex with nonregular non-commercial sex partners in the last month. None of the men who sex with men had ever injected drugs. men who sex with men group had the highest knowledge about HIV; they were aware that using condoms during vaginal sex (85%) or anal sex (96%) reduces risk of HIV transmission. 81% of men who sex with men agreed that having sex with only one partner reduces risk. All of the men who sex with men responded that a condom should be used when having sex.
10 Kassak (2011); Lebanon	Assess the prevalence of, and the risk behaviors associated with the hepatitis B virus and hepatitis C virus infections among two high-risk groups: female sex workers and men who sex with men.	**Design:** Quantitative cross-sectional **Age**: 18 and above **Gender**: Cisgender male and female **Sexual orientation:** Homosexual, heterosexual **Sample size:***N* = 204 participants	None of the participants has been exposed to Hepatitis C virus. In the 101 men who sex with men, only one was Hepatitis B virus antigen carrier and one was confirmed to be anti-HIV positive. 64.8% of men who sex with men had ≥5 occasional partners; 37.2% of men who sex with men reported always using condoms in the past month with occasional partners.
11 Wagner (2012); Lebanon	Explore sexual identity development, sexual risk behavior, and HIV testing among men who sex with men in Beirut, Lebanon.	**Design:** Qualitative study **Sampling:** Purposive sampling **Age:** Mean = 28 **Gender**: Male **Sexual orientation:** Must have had sexual activity with another male in the past year. **Sample Size**: 31 participants	Themes identified: Processes of sexual identity development (comfort with sexual orientation; disclosure of sexual orientation; receipt of support versus stigma in response to disclosure); Sexual and HIV testing; Anxiety and fear about disease.
12 Wagner (2014); Lebanon	Examine the prevalence of, determinants of sexual risk behaviors and testing of HIV infection and other sexually transmitted infections among men who sex with men.	**Design:** Quantitative cross-sectional **Age:** Mean = 26 **Gender:** Cisgender male **Sexual orientation:** Must have had oral/anal sex with a man in the past 12 months **Sample size:***N* = 213	Considering both receptive and insertive anal sex, 70% of the 193 men who had anal sex reported any unprotected anal sex within the past 3 months, of whom 49 had unprotected sex with an HIV-positive or unknown status partner. A total of 116 men reported either unprotected insertive or receptive anal sex during their last anal sex encounter. 62% self-reported ever testing for HIV, and 42% had tested within the past 12 months. A total of 1.5% were considered to be HIV-positive. Two tested positive for Hepatitis B virus and 3 for syphilis. 49% participants reported ever having had a specific sexually transmitted infection, with the most common being genital lice (33%), gonorrhea (27%), chlamydia (19%), genital warts (14%), and human papilloma virus (9%).
13 Maatouk (2014); Lebanon	Report on characteristics of 5 patients with syphilis in a Lebanese hospital	**Design:** Qualitative study **Age:** No specific restriction **Gender:** Cisgender men **Sexual orientation:** Must have had sex with men **Sample size:** N **=** 5	All participants had more than five sexual partners per month. Four participants reported consistent condom use during anal sex and the use of recreational drugs. One participant was co-infected with HIV. All participants had a history of at least one sexually transmitted infection.
14 Aunon (2015); Lebanon	Explore the factors influencing sexual risk behaviors and HIV testing among male sex workers.	**Design:** Qualitative study**Age:** Mean = 24; range: 19–31 **Gender:** Cisgender male **Sexual orientation:** Homosexual, bisexual, and heterosexual**Sample Size:***N* **=** 16	Themes identified: Disclosure of homosexuality and engagement in sex work; condom use with clients and nonclient partners; HIV and testing
15 Kaplan (2015); Lebanon	Explore the risk behaviors of transgender women (trans women) in Lebanon and assess HIV incidence and other social, behavioral, and environmental factors.	**Design**: Qualitative **Age**: Mean = 27; range: 18–54 **Gender:** Transgender women **Sexual orientation:** Heterosexual **Sample size:***N* = 10	Themes identified: Social and emotional safety; physical safety; sexual safety; financial safety.
16 Wagner (2015); Lebanon	Examine the social determinants of sexual risk behavior and HIV testing among men who sex with men in Beirut.	**Design:** Quantitative cross-sectional **Age:** Mean = 26 **Gender:** Cisgender men **Sexual orientation:** Must have had oral/anal sex with a man in the past 12 months. **Sample size:***N* = 213	One participant self-reported being HIV-positive and 2 others tested positive. Considering both receptive and insertive anal sex, 70% of the 193 men who had anal sex reported any unprotected anal sex within the previous 3 months. 62% of respondents self-reported ever testing for HIV, and 42% had been tested within the previous 12 months.
17 Kaplan (2016); Lebanon	Assess the demographic correlates of risk behavior and the prevalence of HIV among trans feminine individuals in Lebanon	**Design**: Quantitative cross-sectional **Age**: Median = 22; range: 18–58 **Gender**: Transgender feminine **Sexual orientation:** Gay, heterosexual, bisexual, and other/unknown categories **Sample size:***N* = 53	92% reported having had receptive anal intercourse with a man in the last three months, most of whom (61%) had condomless receptive anal intercourse (56% of total study sample). Higher sexual activity was associated with condomless receptive anal intercourse. 43% had undergone HIV testing in the past 12 months. Around half of the participants did not know where a free HIV clinic might be accessed. 10% out of the 40 participants who were tested as part of the study or via self-report were HIV positive.
18 Kaplan (2016); Lebanon	Describe the men who sex with men category and the problematic conflation of transgender women and men who sex with men in the existing HIV literature by drawing from examples among trans feminine people in Beirut and San Francisco	**Design**: Qualitative study **Age:** Not specified **Gender:** Transgender female **Sexual orientation:** Not specified **Sample size:***N* **=** 32 participants (10 Transgender women in Lebanon and 22 in San Francisco)	The way participants in Lebanon described their identity indicates that this terminology is complex across different contexts and HIV risk categories have problems. In non-Euro-Atlantic contexts, positive familial relationships impact an individual's desire and motivation to ‘transition’ either medically/socially. This impacts HIV risk and vulnerability to other outcomes.
19 Tohme (2016); Lebanon	Investigate the prevalence and correlates of HIV testing and condom use among Iraqi, Syrian, and Palestinian men who sex with men refugees in Beirut, Lebanon	**Method:** Quantitative cross-sectional **Age**: Mean = 27; range: 18–40 **Gender:** Cisgender male **Sexual orientation**: Homosexual **Sample size:***N* **=** 150	76.6% of the overall participants reported condomless receptive sex and 59.3% reported condomless receptive sex with a male partner who was HIV-positive or had an unknown HIV status. Of those who had insertive anal sex, 84.6% reported condomless sex and 42% reported condomless insertive sex with a male partner who was HIV-positive or had an unknown HIV status. 48% reported ever having been tested for HIV. 60.7% reported ever testing for any sexually transmitted infections. Participants who saw a medical doctor in the previous year had 6 times greater odds of having an HIV test prior to the study; those who knew where to find HIV testing had less odds of reporting any unprotected anal intercourse regardless of partner status and greater odds of ever having been HIV tested.
20 Tohme (2016); Lebanon	To explore HIV transmission and sociodemographic correlates of condom use and HIV testing among men who sex with men refugees in Lebanon.	**Method:** Quantitative cross-sectional **Age**: Mean = 27 **Gender:** Cisgender male **Sexual orientation**: Homosexual **Sample size:***N* = 150	48.0% self-reported ever testing for HIV. A total of 2.7% were considered to be HIV positive. 60% participants reported ever having had a specific sexually transmitted infections, with the most common being human papilloma virus /genital warts (28.7%), gonorrhea (24%), chlamydia (23.3%), and genital lice (18%). Relationship status, sex work, and self-identifying as gay, and spending fewer years in Lebanon were significant predictors of HIV testing.
21 Maatouk (2016); Lebanon	Assess human papilloma virus prevalence in the oral cavity of men who sex with men from Beirut, Lebanon.	**Design:** Quantitative cross-sectional **Age:** 18 years or older **Gender:** Cisgender male **Sexual orientation:** Gay, bisexual, or heterosexual, and had sex with a man in the past 6 months. **Sample size:***N* = 42	33.33% were HIV-positive; 55% had a history of genital/anal warts and 30.9% reported a previous sexually transmitted infection. Moreover, 64% of HIV-positive and 35% of HIV-negative participants used a condom at last anal intercourse. Human papilloma virus prevalence in the oral cavity was 10%. The human papilloma virus type was exclusively human papilloma virus −6.
22 Maatouk (2016); Lebanon	Assess sexual behaviors among men who have sex with men (men who sex with men) who attended a dermatology-sexually transmitted infection clinic.	**Design:** Quantitative cross-sectional **Age:** No restrictions were reported **Gender:** Cisgender men **Sexual orientation:** Must have had sex with a man **Sample size:***N* = 31	58.1% participants had unprotected anal sex; 9.7% tested positive for HIV; 71% had undergone at least one sexually transmitted infection test in the past year; 45.2% had 2–5 sexual partners for the last 6 months; and 42.9% of our participants reported a history of testing positive for Syphilis.
23 Heimer (2017); Lebanon	Report on HIV prevalence and identify HIV risk behaviors and psychological factors associated with prevalent infection among men who sex with men	**Design**: Quantitative cross-sectional **Age**: Mean = 27 **Gender:** Cisgender male **Sexual orientation:** Homosexual **Sample size:***N* = 292	Only seven of men who reported having sex at least 10 times in the past year with a single partner reported always using condoms. Prevalence of HIV was estimated at 13.0% among the 276 participants with any history of testing and 14.9% among those who reported a prior test and either were positive at that test or tested negative then and agreed to be retested as part of this study (*n* = 242). Sociodemographic factors associated with HIV-positive status included older age, Lebanese birth, and higher income.
24 Gereige (2018); Lebanon	Compare the sexual health of sexual minority women and heterosexual women living in Lebanon.	**Design**: Quantitative cross-sectional **Age**: 18 years and older **Gender:** Cisgender women **Sexual orientation:** Heterosexual, bisexual, lesbian, and other (queer). **Sample:***N* = 95 participants	Sexual minority women were on average 19 years at their sexual debut with men, significantly younger than heterosexual women's age of 21. Combining both male and female partners, sexual minority women had a significantly higher number of lifetime sex partners of either sex. More than 50% of sexual minority women reported some form of unwanted sexual contact in their lifetime, compared to 23% of heterosexual.
25 Assi (2019); Lebanon	Assess the prevalence of HIV and sexually transmitted infections among men who have sex with men in Lebanon.	**Design:** Quantitative cross-sectional **Age:** Mean = 26; range: 15–69 **Gender:** Cisgender man **Sexual orientation:** Men who sex with men **Sample Size:***N* = 2238	Prevalence of sexually transmitted infections: HIV: 5.6%; human papilloma virus: 41.0%; gonorrhea and/or chlamydia: 17.5%; syphilis: 3%. Condom use: Majority had inconsistent condom-use (67%), 78% had more than one sexual partner in the last three months. The majority reported unprotected oral and anal sex exposures in the past three months (99% and 53% respectively).
26 Kaplan (2019); Lebanon	Explore the feasibility and acceptability of the adapted intervention TransAction (Baynetna), an HIV prevention intervention for transgender women	**Design:** Mixed-methods **Age**: Median = 26; range: 22–50 **Gender**: Transgender women **Sexual orientation:** Not specified **Sample size:***N* = 16	Baynetna, a behavioral group support trans-delivered intervention, was feasible and acceptable in Lebanon.
27 Storholm (2019); Lebanon	Examine socioecological factors associated with willingness to take pre-exposure prophylaxis among a cohort of young men who sex with men from Beirut, Lebanon	**Method:** Quantitative cross-sectional **Age**: 18 – 29 years **Gender:** Cisgender male **Sexual orientation**: Homosexual **Sample size:***N* = 218	Most young men who sex with men were willing to take pre-exposure prophylaxis and claimed that it was beneficial. 99 (45.4%) said it was very likely, 22 (10.1%) said somewhat likely, 59 (27.1%) said somewhat unlikely, and 38 (17.4%) said it was not at all likely they would use pre-exposure prophylaxis.
28 Ghanem (2020); Lebanon	Examine the relationships between measures of sexual identity development and HIV protective behaviors.	**Design**: Quantitative cross-sectional **Age**: Mean = 24; range: 18–29 **Gender:** Cisgender male **Sexual orientation:** Homosexual **Sample:***N* = 218	48.9% of the 176 men who had anal sex reported any condomless anal sex within the past 3 months, of whom 17.0% had such sex with an HIV-positive (*n* = 4) or unknown status (*n* = 26) partner. 81.7% reported being tested for HIV in their lifetime, but 50.9% had been tested within 6 months prior to the survey. Greater integration into the gay community was the only measure of sexual identity development that was significantly correlated with having any condomless anal sex with partners or ever having been tested for HIV.
29 Kaplan (2020); Lebanon	To pilot test an adapted intervention, ‘‘Baynetna’’ to assess preliminary impact on mental and sexual health of transgender women.	**Design**: Mixed-methods experimental design **Age:** Median = 26 **Gender:** Transgender women **Sexual orientation:** Not specified **Sample size:***N* = 16	At the 6-month post-test, 7 out of 13 participants had improved gender affirmation satisfaction scores compared with baseline; 9 out of 13 participants had improved community connectedness scores compared with baseline; and 9 out of 13 participants had improved social cohesion scores. There was an increase in proportion of participants testing for HIV between the 6 months before enrollment and the 6 months after enrollment.
30 Wagner (2020); Lebanon	Describe the sexual risk and HIV testing behaviors of young men who sex with men in 2017, and how they compare to the same behaviors reported in 2012.	**Design:** Quantitative cross-sectional **Age:** Mean = 23; range: 18–29 **Gender:** Cisgender male **Sexuality**: Homosexual **Sample size:** in 2012, *N* = 164; and in 2017, *N* = 226	**Sexual behaviors in past 3 months** Compared to the 2017 study sample, a similar proportion of the 2012 sample reported engagement in receptive anal sex in the prior 3 months, while a smaller proportion reported insertive anal sex. The 2017 sample was about half as likely to have had any condomless anal sex in the past 3 months, including with partners whose HIV status was positive or unknown, compared to men in the 2012 sample. The 2017 was nearly two and a half times more likely to have had an HIV test within the past 6 months compared to the 2012 sample; 1% 2012 self-reported being HIV positive, compared to 3.6% in 2017.
31 Maatouk (2020); Lebanon	Present data on the prevalence of sexually transmitted infections in a sample of men who sex with men attending a sexual health clinic	**Design:** Quantitative cross-sectional **Age:** No restrictions were reported **Gender:** Cisgender male **Sexual orientation:** Must have had sex with a man **Sample size:***N* = 1364	Most frequent sexually transmitted infections were: genital warts (41.13%), Chlamydia (25.85%), anogenital herpes (24.49%), Neisseria gonorrhea (22.87%), syphilis (13.41%), scabies/pediculosis (10.19%), HIV (9.5%), Mycoplasma genitalium (4.88%), Hepatitis B (0.73%), lympho-granuloma venereum (0.45%), and Hepatitis C (0.09%).
32 Valadez (2013); Libya	Provide baseline information on the level of HIV infection and related socio-demographic, behavioral and other characteristics among men who sex with men and female sex workers.	**Method:** Cross-sectional quantitative **Age**: 15 years and older **Gender:** Cisgender male and female **Sexual orientation**: Homosexual and heterosexual **Sample size:***N* = 541	For men who sex with men, HIV prevalence was 3.1%, Hepatitis B virus prevalence was 2.9%, and Hepatitis C virus was prevalence 7.3%. 2.1% participants were HIV/ Hepatitis C virus co-infected. 45.6% of men who sex with men had been tested for HIV during the past year. 21% of men who sex with men used a condom during last anal intercourse; 26.5% had anal intercourse with a commercial partner in the past six months, but only 19.4% of these used a condom at last commercial intercourse. Only 16.8% of men who sex with men succeeded in both, correctly identifying ways to prevent sexual HIV transmission and rejecting major misconceptions about HIV transmission. sexually transmitted infections-related knowledge was very poor.
33 Johnston (2013); Morocco	Report on HIV and syphilis prevalence and sexual risk behaviors among men who have sex with men (men who sex with men) in Agadir and Marrakech, Morocco.	**Design**: Quantitative cross-sectional **Age:** Most participants were < 25 years of age. **Gender**: Cisgender men **Sexual orientation:** Bisexual, homosexual, heterosexual **Sample size:***N* = 669	64.7% and 65.6% of men in both cities (Marrakech and Agadir) reported receiving money for sex. Condom use at last anal with occasional male partner was 49.3% in Agadir and 37.1% in Marrakesh. men who sex with men in Agadir had a higher HIV positive prevalence (5.6% versus 2.8%), but lower syphilis prevalence (7% and 10.8%), compared with men who sex with men in Marrakesh. Co-infection with HIV and syphilis was 31.6% in Agadir and 56.4% in Marrakesh.
34 Bokhari (2007); Pakistan	To measure HIV prevalence and risk behavior in injecting drug users, male sex workers, Hijras (transgenders), female sex workers, and male truckers in Karachi and Lahore, Pakistan	**Design:** Quantitative cross-sectional **Age:** No age restrictions **Gender:** Male, female, and male to female transgender participants **Sexual orientation:** Not specified **Sample Size:** 3640 (400 participants in each city were chosen for every group besides Hijras (sample size of 200 participants)	**HIV prevalence and risk behaviors:** HIV prevalence was 2% among Hijras. Hijras reported a median duration of commercial sex experience of six years in Lahore and 10 years in Karachi; 96% had sold anal sex to men in the past month, with a median of four partners in the past week (range 1–21). Forty percent (40%) had non-paying male anal sex partners in the past month, and they used a condom during 8% of last sex acts with these partners. Eleven percent (11%) had sex with a woman in the last year, and 25% of last female sex acts were covered by a condom. Intervention contact in the past year was below 2% for male sex workers, Hijras and truckers. Contact was defined as having been specifically approached by someone for the purpose of discussing HIV prevention or having attended a meeting for HIV prevention.
35 Collumbien (2008); Pakistan	Examine risk behaviors among male and transgender sex workers by exploring the context of their relationship with clients who are injecting drug users.	**Design:** Mixed-methods **Age:** Not specified **Gender:** Cisgender male and transgender participants **Sexual orientation:** Homosexual **Sample Size:***N* **=** 30 for qualitative interviews; *N* = 918 for quantitative phase	35% of sex workers with injecting drug users clients used a condom at last sex with a client compared to 26% of sex workers with no injecting drug users clients; 73% either sometimes or always used condoms compared to 48% with no injecting drug users clients. Knowledge level of HIV/AIDS and its transmission was extremely low in both group of sex worker (with and with no injecting drug users clients): 21% and 42% ever having heard of HIV, 7% and 6% had ever been tested for HIV, respectively.
36 Khan (2008); Pakistan	Explore sexual behaviors, relationships and prevalence of HIV and sexually transmitted infections among Hijras	**Design**: Quantitative cross sectional **Age:** Mean = 24 **Gender:** Transgender **Sexual orientation:** Sex with men, women, and other transgender individuals **Sample size:***N* = 409	Sexual behaviors: 15% reported condom use at last sex encounter; 83% had never asked a client to use condoms; 89% never used condoms with non-paying clients. Sexually transmitted infections prevalence: participants had a history of one (59%) or more (38%) sexually transmitted infections. sexually transmitted infections/HIV knowledge: 68% had heard of HIV and associated risk reduction with condom use (69%), avoidance of anal sex (73%) or needle sharing (87%).
37 Saleem (2008); Pakistan	Investigate the sociodemographics, risky sexual behaviors, knowledge of HIV and sexually transmitted infections, and treatment seeking behaviors among female sex workers, male sex workers, Hijras, and injecting drug users	**Methods:** Quantitative cross-sectional **Age**: Mean = 26 **Gender**: Cisgender male, female, and transgender **Sexual orientation**: Not specified **Sample Size:** 605	Mean ages of first intercourse for Hijras was 13.9 years of age. Consistent condom use among hijras was 4% sexually transmitted infections knowledge among Hijras was 59.4%; HIV knowledge was 78%. 24% had a history of sexually transmitted infections in last six months; 88% sought treatment for sexually transmitted infections.
38 Hawkes (2009); Pakistan	Understand the spread of the HIV epidemic in Pakistan among men, women and transgender populations selling sex in Pakistan	**Design**: Quantitative cross-sectional **Age**: Mean = 30 **Gender:** Cisgender male, female, and transgender **Sexual orientation:** Homosexual; heterosexual **Sample size:***N* = 1455	29% of transgender and 20% of male sex workers reported recent symptoms of sexually transmitted infections. Numbers of clients and new clients was higher among male and transgender sex workers compared to female sex workers. Male and transgender sex workers had consistently low rates of condom use.
39 Khanani (2010); Pakistan	Assess HIV, Hepatitis C virus, and Hepatitis B virus among the men who sex with men community.	**Design:** Quantitative cross-sectional **Age:** 40% were 21–30 years of age **Gender:** Cisgender male **Sexual orientation:** Homosexual **Sample size:***N* = 396	A total of 20 (5.05%), 66 (16.66%), and 23 (5.80%) participants were infected with, respectively, HIV, Hepatitis C virus, and Hepatitis B virus alone. Twenty participants (5.05%), 2 (0.50%), and 4 (1.01%) were co-infected with, respectively, HIV- Hepatitis C virus, HIV- Hepatitis B virus, and Hepatitis C virus - Hepatitis B virus. Three (0.75%) participants were infected with all the three viruses.
40 Khanani (2011); Pakistan	Explore the transmission pattern of HIV among Injecting Drug Users and Men who have Sex with Men, their spouses and children.	**Design:** Quantitative cross-sectional **Age:** Range: 18–46 **Gender:** Cisgender male **Sexual orientation:** Men who sex with men **Sample size:** 47 men who sex with men, 15 men who sex with men spouses, and 14 men who sex with men children	There was bridging of HIV infection from the core high risk group of injecting drug users, men who sex with men, and men who sex with men-injecting drug users. Men who have sex with men-injecting drug users may bridge HIV transmission between injecting drug users and men who sex with men through needle sharing.
41 Shaw (2011); Pakistan	Describe and compare characteristics of Hijra sex workers and male sex workers from eight cities in Pakistan.	**Design**: Quantitative cross-sectional **Age**: Mean = 24 **Gender:** Transgender, and cisgender male **Sexual orientation:** Not specified **Sample:***N* = 2694	Risk sexual behavior: Hijra sex workers were less likely to report condom use at last anal sex than male sex workers. HIV prevalence for Hijra sex workers was 0.6%, while for male sex workers it was 0.4%. Condom use: Married sex workers were more likely to report condom use; greater numbers of anal sex clients were negatively associated with condom use; reported sex with a known injecting drug user was positively associated with condom use.
42 Akhtar (2012); Pakistan	Investigate prevalence of HIV infection among high-risk transgender men.	**Design:** Quantitative cross-sectional **Age:** Median = 29; range: 15–64 **Gender:** Transgender males-Hijras (defined by authors as “males by nature but appearing as women”) **Sexual orientation:** Homosexual and heterosexual orientations **Sample Size:***N* **=** 306	HIV prevalence was 21.6%. Older transgender males (>30 years of age) were at higher risk of having HIV, compared to those <30 years of age.
43 Altaf (2012); Pakistan	Compare sexual risk behaviors Hijra Sex Workers in Lakarna and other cities of Pakistan.	**Design:** Quantitative cross-sectional **Age:** Mean = 26; range: 15–45 **Gender:** Transgender **Sexual orientation:** Homosexual **Sample Size:***N* **=** 619	Factors associated with higher prevalence of HIV in Larkana: younger age 20–24 years, being unmarried, mode of income only sex work, and duration in sex work 5–10 years or more. More participants in Larkana compared to other cities believed that HIV can also spread through blood transfusion; majority of participants in Larkana did not believe that prevention of HIV/sexually transmitted infections is possible by refraining from sex. Significantly lower number of participants in Larkana than other cities used condom during sexual intercourse; 12.7% of participants were ever tested for HIV.
44 Reza (2013); Pakistan	Explore past and future HIV trends among injecting drug users, male sex workers, hijra (transgender; Hijra sex workers) and female sex workers.	**Design**: Quantitative cross-sectional **Age**: Male sex workers 13 years and older; Hijra sex workers and female sex workers s 15 years and older; injecting drug users 18 years and older **Gender**: Cisgender male, cisgender female, and transgender participants **Sexual orientation:** Not specified **Sample size:** 7118 injecting drug users, 6638 female sex workers and 10,760 male sex workers/Hijra sex workers	HIV prevalence among male sex workers/Hijra sex workers was higher than among female sex workers, but lower than among injecting drug users; HIV prevalence among male sex workers/Hijra sex workers increased over time to reach 14% in 2008.
45 Mir (2013); Pakistan	Assess the range and magnitude of urban men's non-marital sexual behaviors, focusing on men having sex with men.	**Design:** Quantitative cross-sectional **Age:** Mean = 29; range: 16–45 **Gender:** Cisgender male **Sexual orientation:** Bisexual and homosexual men **Sample size:***N* **=** 2400	211 participants had sex with men (26% were married; 62% exclusively had sex with males). Nearly half of the men who sex with men reported having had three or more sexual partners in the last 12 months and nearly half considered themselves to be at risk of acquiring a sexually transmitted infection.
46 Melesse (2016); Pakistan	Assess the heterogeneity in overlapping HIV risk behaviors among sex workers.	**Design:** Quantitative cross-sectional **Age:** Mean = 26 **Gender:** Cisgender male, female, and transgender **Sexual orientation:** Not specified **Sample size:***N* = 8483	Overlapping risk behaviors (both injecting drugs and having sex with people who inject drugs) were the highest among Hijra sex workers (2.99%) followed by female sex workers (2.25%) and male sex workers (1.20%). Exposure to at least one risk behavior, either injecting drugs or having sex with a person who injects drugs, was highest among female sex workers (14.32%) followed by Hijra sex workers (13.55%) and male sex workers (12.24%). Condom use: 45.55% of sex workers used a condom in the last sexual intercourse with any paying client, and this varied from 53.47% among female sex workers to 31.87% and 22.87% among Hijra sex workers and male sex workers, respectively. In general, those who injected drugs were less likely to use a condom with paying clients. HIV prevalence was the highest among Hijra sex workers (8.14%), followed by 3.57% among male sex workers and 0.97% among female sex workers.
47 Hasan (2018); Pakistan	Investigate the recency of HIV infections in newly-diagnosed cases in people who inject drugs and transgender Hijra sex workers.	**Design**: Quantitative cross-sectional **Age**: Mean = 30 **Gender:** Hijra/Transgender (gender for people injecting drugs was not specified) **Sexual orientation:** Have sex with men **Sample size:** 210 participants	18% of individuals had recently-acquired HIV infections and 82% had late HIV infections. Nineteen percent of people who inject drugs had recent and 81% had late infections; 17% of transgender Hijra sex workers had recent and 83% had late, chronic HIV infections.
48 Usman (2018); Pakistan	Examine the perceptions and experiences of HIV infected Hijra Sex Workers.	**Design:** Qualitative **Age**: Range: 18–55 **Gender:** Transgender women (Hijra) **Sexual orientation:** Not specified **Sample size:***N* = 16	Themes identified: Post-diagnosis identity and body image; sexual conduct and clientage process; religious and spiritual life; and social support system.
49 Melesse (2018); Pakistan	Explore disparities in geographical trends of HIV epidemics among people who inject drugs, female sex workers and hijra/transgender/male sex workers (H/male sex workers)	**Design:** Quantitative cross-sectional **Age:** Mean age of people who inject drugs: 33; female sex workers: 27; and Hijra/male sex workers: 25 **Gender:** Cisgender male, female, and transgender **Sexual orientation:** Not specified **Sample size:***N* = 43,522	HIV prevalence estimates: More than half of people who inject drugs (54.7%, plausible range: 52.4–56.9%) and nearly one-fifth of Hijra sex workers and male sex workers (19.6%, plausible range: 17.7–22.4%) may be living with HIV by 2020. HIV incidence estimates: Estimated incidence among Hijra sex workers and male sex workers has increased from 9.1 new infections per 1000 person-years (plausible range, 8.6–10.0) in 2010 to 18.9 (plausible range, 16.7–22.0) in 2015 and projected to continue rising to 24.3 (plausible range, 20.7–28.3) by 2020.
50 Khalid (2019); Pakistan	Explore relationship between network operators and risky sex behaviors among female and transgender commercial sex workers in four Pakistani provinces.	**Design**: Quantitative cross sectional **Age:** Mean = 27 **Gender:** Cisgender female and transgender **Sexual orientation:** Not specified **Sample size:***N* **=** 2326	Condom use: 45% of transgender sex workers used condoms consistently. Network operators: more cisgender females (46%) than transgender (21%) sex workers recruited clients through network operators. Transgender sex workers who use network operators are at higher odds to use condoms consistently than cisgender female sex workers
51 Mirzazadeh (2011); Yemen	Estimate the prevalence of HIV and related risk behaviors among men who sex with men in Yemen	**Design:** Quantitative cross-sectional **Age:** Mean = 24 **Gender:** Cisgender male **Sexual orientation:** Gay, bisexual, or heterosexual, and had sex with a man in the past 6 months **Sample size:***N* = 261	88.0% of participants reported engaging in insertive anal sex partner and 78.1% reported receptive anal sex. The average number of male sexual partners during the last 6 months was 3.8 (insertive anal sex) and 5.7 (receptive anal sex). 20% of participants used condoms during the last anal sex. men who sex with men who had never used condoms with any partner, ranged from 63.9% for those with commercial partners to 76.4% for those with casual partners. 89.6% of men who sex with men had heard of sexually transmitted infections. Sexually transmitted infections symptoms in the last 12 months were reported by 26.9% of participants; 67.1% did not seek treatment. 98.8% of men who sex with men had heard of HIV/AIDS, but 27.8% had comprehensive knowledge. 36.2% of participants had ever tested for HIV. 5.7% were HIV positive.
52 Matarelli (2013); Multiple MENA countries	To investigate Sexual Sensation Seeking Scale scores to predict numbers of recent men who sex with men sexual activities and to predict any recent unprotected receptive anal intercourse activities.	**Design:** Quantitative cross sectional **Age:** 18 years or older **Gender:** Cisgender male **Sexual orientation:** Men who sex with men **Sample size:***N* = 86	34.9% of participants reported 1 or more unprotected receptive anal intercourse activities. 53.5% have taken an HIV test in the past. Higher Sexual Sensation Seeking Scale scores predicted higher numbers of recent men who sex with men sexual activities and unprotected receptive anal intercourse.

Studies are listed chronologically in alphabetical order of countries; HIV: human immunodeficiency virus; AIDS: acquired immunodeficiency syndrome.

**Table 3:** Mental health (*n* = 20); Studies about gender (*n* = 17); Violence and discrimination (*n* = 7); Experiences with healthcare system (*n* = 2).

Mental health			
First author (Year); Country	Study aims	Study design and sample characteristics	Relevant findings
1 Aghbikloo (2012); Iran	Assess the socio-demographic characteristics and mental health of Iranian transsexual individuals applying for sex reassignment surgery.	**Design:** Quantitative cross sectional **Age**: Mean = 25 **Gender:** Transgender (male-to-female and female-to-male) **Sexual orientation:** Not specified **Sample size:***N* = 69	Among all participants, 69.6% reported onset of symptoms in childhood, 18.8% in puberty, and 11.6% had late onset of symptoms. Among female-to-male: 36% had anxiety disorders; 24% mood disorders, and 4% substance related disorders. Among male-to-female: 29.5% had mood disorders; 27.3% substance-related disorders; 6.8% anxiety disorders; and 2.3% schizophrenia.
2 Meybodi (2014); Iran	Assess the frequency of personality disorders in Iranian gender identity disorder patients	**Design:** Quantitative cross-sectional **Age:** No age restrictions **Gender:** Biologically male and female **Sexual orientation:** Not specified **Sample size:***N* = 73	The most frequent personality disorder was narcissistic personality disorder (57.1%) and the least frequent was borderline personality disorder. The average number of diagnoses was 3.00 per patient. Schizoid, schizotypal, and avoidant personality disorders and passive-aggressive and dependent personality disorders were significantly more prevalent in biological males than biological females.
3 Nematy (2014); Iran	Compare the Early Maladaptive Schemata in homosexual and bisexual people and compare them with heterosexual people.	**Design:** Quantitative cross-sectional **Age**: Mean = 26 **Gender:** Cisgender men and women **Sexual orientation:** Homosexual, bisexual, heterosexual **Sample size:***N* = 150	Homosexual participants had significantly higher scores than heterosexual participants on the following: mistrust/abuse, social isolation, defectiveness/shame, sacrifice and emotional inhibition. Individuals whose families were not aware of their sexual orientation scored significantly higher on the social isolation schema, vulnerability to harm or illness, emotional inhibition, and unrelenting standards than individuals whose families were informed.
4 Havar (2015); Iran	Survey the rate of prevalence of comorbid psychiatric and personality disorders among individuals with Gender Identity Disorder	**Design:** Quantitative cross-sectional **Sampling:** Convenience **Age**: Range: 15–40 **Gender:** Transgender men and women **Sexual orientation:** Not specified **Sample size:***N* = 108	20.4% individuals had depressive personality disorder, 1.9% had paranoia personality disorder, 6.5% had histrionic disorder, 12.0% had obsessive- compulsive personality disorder, 4.6% had narcissistic personality disorder, 2.8% had dependent personality disorder, 5.6% had schizotypal personality disorder, 1.9% had passive-aggressive personality disorder, and the rest 23.1% had personality disorders (not specified). There was a significant difference between male-to-female and female-to-male groups in terms of prevalence of personality disorders.
5 Nematy (2016); Iran	Compare the attachment style of sexual minorities and their heterosexual counterparts	**Design:** Quantitative cross-sectional **Age**: Mean = 26 **Gender:** Cisgender men and women **Sexual orientation:** Homosexual, bisexual, heterosexual **Sample size:***N* = 150	Lesbian, gay and bisexual people were significantly more anxious and less dependent than the control group. Lesbian, gay and bisexual persons who are not satisfied with their sexual orientation are less dependent and more anxious than satisfied ones.
6 Fallahtafti (2019); Iran	Investigate happiness and mental health in transsexual patients pre/post-surgery.	**Design**: Quasi-experimental study **Age:** Mean = 25 **Gender:** Transsexual (both female-to-male and male-to-female) **Sexual orientation:** Not specified **Sample size:***N* = 66	There is significant difference between the mean score of pre-operative and post-operative transsexual individuals on the following: happiness; mental health; somatization; anxiety; depression; interpersonal sensitivity; phobia; obsession-compulsion; paranoia; and psychosis.
7 Asadi (2020); Iran	Investigate the effect of empowerment model-based training on the quality of life of transgender people undergoing hormone therapy.	**Design:** Randomized clinical trial **Age:** Range: 18–35 **Gender:** Transgender **Sexual orientation**: Not specified **Sample size:***N* = 81	Post intervention: intervention group had significantly better quality of life, perception of health, mental health, emotional and overt anxiety than control group.
8 Shirdel-Havar (2019); Iran and The Netherlands	Compare the mental health of transgender individuals from an Islamic and non-Islamic country (Iran and the Netherlands).	**Design:** Quantitative cross-sectional **Age**: Iranian sample (Mean = 25). **Gender:** Transgender men and women **Sexual orientation: N**ot specified **Sample size: T**otal *N* = 163 (120 in the Netherlands and 43 in Iran)	Iranian participants were more often bisexual than the Dutch participants. They also had significantly more psychological symptoms and had higher Gender Dysphoria scores than the Dutch participants. Significantly more Dutch participants were married, had more contact with their families, were more dissatisfied with their secondary sexual characteristics and neutral body characteristics than the participants in Iran.
9 Scull (2017); Kuwait	Investigate the lived experiences of Arab sexual minorities living in conservative Islamic country	**Design:** Qualitative (phenomenology) **Age**: Mean = 25; range: 20–32 **Gender:** Cisgender men and women **Sexual orientation:** Gay, lesbian, bisexual **Sample size:***N* = 10	Themes identified: The role of culture and religion; risks; coping by developing community; political factors.
10 Wagner (2013); Lebanon	Explore sexual identity development among men who have sex with men in Beirut, Lebanon	**Design:** Qualitative study **Age:** Mean = 28; range: 19–65 **Gender**: Male **Sexual orientation:** Homosexual and bisexual **Sample size:***N* = 31	Themes identified: Relationship with and disclosure to family; Disclosure to friends and co-workers; Experiences of stigma and discrimination; Negative health effects; Coping with stigma.
11 Ibrahim (2016); Lebanon	Assess psychiatric comorbidity in a population of Lebanese transgender individuals and compare it to the general population.	**Design:** Quantitative comparative study **Age:** Mean = 24 **Gender:** Transgender and cisgender **Sexual orientation:** Not mentioned **Sample size:***N* = 40	Fifty-five percent of transgender participants had active suicidal thoughts compared to 0% among cisgender participants. Among transgender participants: 45% had a major depressive episode, 5% had generalized anxiety disorder, 5% had post traumatic stress disorder, and 10% had major depressive episode with comorbid posttraumatic stress disorder.
12 Kaplan (2016); Lebanon	Examine risk factors associated with suicide attempts among trans feminine individuals in Beirut, Lebanon.	**Design**: Quantitative cross-sectional **Age**: Mean = 27; range: 18–58 **Gender:** Transgender women **Sexual orientation:** Heterosexual **Sample size:***N* **=** 54	Prevalence of depression and suicide attempts: 70% of participants scored greater than 2 on the PHQ-2; 66% of participants scored greater than 9 and were classified as currently depressed. 39% of participants reported never having suicidal thoughts, 15% had only passing thoughts of suicide, 6% had a suicidal plan at least once but never attempted to carry it out, and 46% reported having attempted suicide. Correlates of suicide: History of attempted suicide was significantly associated with lower general social support, lower social integration, and lower support from peers, being more open about transgender identity in public, and any hormone use (past or current).
13 Mutchler (2018); Lebanon	Examine internal dynamics within Beirut's gay community as a basis for developing community-level interventions.	**Design:** Qualitative **Age:** Range: 18–29 **Gender:** Male **Sexual orientation:** Gay or had sex with a man **Sample size:** 25–35 (5 focus groups with 5–7 participants in each)	Themes identified: Importance of safe space: access to spaces; Finding each other: balancing safety with visibility; The struggle for support: community divisions, barriers and facilitators to connecting.
14 Wagner (2019); Lebanon	Assess depression and its relationship to structural and sexual minority-related stressors and social support in men who have sex with men	**Design**: Quantitative cross-sectional **Age**: Mean = 24 **Gender:** Cisgender male **Sexual orientation:** Must have had oral or anal sex with a man in the past 12 months **Sample size:***N* = 226	40.7% met the definition of clinical depression and 15.9% met criteria for major depression using the Patient Health Questionnaire-9. 33.2% reported any lifetime history of suicidal ideation; 14.7% reported any lifetime history of having had a plan to attempt suicide. Structural stressors included unemployment (41.6%) and not having legal residency status (13.7%). Sexual minority-related stressors consisted of internalized stigma and discrimination. 29.2% were somewhat comfortable to very uncomfortable with their sexual identity. 69.0% had experienced at least one type of discrimination in the past year.
15 Michli (2020); Lebanon	Investigate internalized homonegativity by exploring risk and protective factors in Lebanese sexual minorities.	**Design:** Quantitative cross-sectional **Age**: Mean = 26 **Gender:** Cisgender men and women **Sexual orientation:** Lesbian, gay, bisexual, queer, straight, and questioning **Sample size:***N* = 210	On average, participants endorsed low levels of internalized homonegativity. Internalized homonegativity was significantly positively correlated with parental rejection, religiosity, and vigilance, and significantly negatively correlated with sense of belonging to the lesbian, gay and bisexual community and self-compassion.
16 Abdullah (2012); Pakistan	Identify factors that cause the Hijra (transgender) community in Rawalpindi to seek commercial sex work, and to assess social exclusion and its impact on Pakistani society.	**Design:** Qualitative **Age:** Range: 16–68 **Gender:** Transgender **Sexual orientation:** Not specified **Sample Size:***N* = 36 participants	Themes identified: Childhood and early life: realization of a difference; A new beginning: the world of hijras; Life as a hijra: away from the mainstream; Khusrapan vs. Zananapan: two schools of thought; Living environments and social contacts; Exclusion from occupational opportunities; Future aspirations: chained dreams; Death in isolation: going away quietly
17 Saeed (2018); Pakistan	Determine the patterns of substance addiction among the transgender community.	**Design:** Qualitative **Age**: Range: 20–57 **Gender:** Transgender **Sexual orientation: N**ot specified **Sample size:***N* = 27	Themes identified: Twofold selves (the dilemma of transgender individuals of living in an in-between state of mind); The Trans world; Torture and harassment experiences in school; Tobacco smoking; Substance abuse; Role of transgender companions in substance abuse; Discrimination in the health sector
18 Azeem (2019); Pakistan	Record the prevalence of suicidal ideation in the transgender population and assess the relationship of depression with the suicidal ideation.	**Design:** Quantitative cross-sectional **Age:** Mean = 39 **Gender:** Transgender **Sexual orientation:** Not specified **Sample Size:***N* = 156	42.9% of participants had suicidal ideation and 63.5% had depression. Depression and illicit substance use were strongly associated with the presence of suicide ideation among transgender individuals.
19 Zubair (2019); Pakistan	Determine the prevalence and correlates of suicidal attempts among the transgender population of twin cities of Pakistan.	**Design**: Quantitative cross sectional **Age:** Mean = 38 **Gender:** Transgender **Sexual orientation**: Not specified **Sample size:***N* = 148	29.1% of participants had one or more suicidal attempts during their life. 8.1% had more than one suicidal attempt while 12.8% had an attempt in last one year. 62.8% of participants had depression. Depression and low income were significantly related to suicidal attempt.
20 Akhtar (2020); Pakistan	Assess mental health among transgender individuals in Pakistan	**Design:** Quantitative cross-sectional **Age:** Mean = 28; range: 19–50 **Gender:** Transgender **Sexual orientation:** Not specified **Sample Size:***N* = 100	29% of the sample showed low level of psychological resilience; 30% showed low levels of self-esteem. Transgender individuals residing with their gurus had a significantly higher level of psychological resilience as well as self-esteem as compared to those living alone or with friends. Education had a significant positive correlation with resilience and self-esteem. A high level of psychological resilience was associated with a high level of self-esteem.
**Studies About Gender**			
**First Author (Year); Country**	**Study Aims**	**Study Design and Sample Characteristics**	**Relevant Findings**
1 Hedjazi (2013); Iran	Describe socio- demographic characteristics in a population of transsexual individuals	**Design:** Quantitative cross-sectional **Age**: Mean = 28 **Gender**: Transsexual **Sexual orientation:** Not specified **Sample size**: *N* = 44	The majority of patients were diploma and higher diploma education (77.3%), lived in urban areas (81.8%), were employed (56.9%), single (93.1%), and under six months of hormonal treatment (61.4%).
2 Alavi (2015); Iran	Identify masculine and feminine gender roles in Iranian patients with gender identity disorder and compare these roles with two control groups	**Design:** Quantitative cross-sectional **Age:** Mean = 31 **Gender: M**ale-to-female transgender, female-to-male transgender, cisgender male and female participants. **Sexual orientation:** Not specified **Sample Size:***N* = 209	Highest and lowest mean scores of Gender Masculine scale belonged to the female-to-male and male-to-female groups, respectively. On the Gender Feminine scale, the female-to-male group had the lowest scores. female-to-male have dominant masculine gender role, while male-to-female have dominant feminine gender role. female-to-male scores were similar to female controls. No one was considered androgynous.
3 Cohanzad (2016); Iran	Introduce the ‘‘extensive metoidioplasty’’ technique for transsexual patients to achieve an effective reconstruction of external male genitalia.	**Design:** Mixed-methods **Age:** Range:20–40 **Gender: F**emale-to-male transsexual patients **Sexual orientation:** Not specified **Sample Size:***N* **=** 10	During a postoperative period of 68.4 months: a mean penile length of 8.7 cm, capable of obtaining erections, enough for intromission in 70% of patients.
4 Valashany (2018); Iran	Evaluate self-reported perceived quality of life in female to male (female-to-male) and male to female (male-to-female) transgender participants and compared to the general population.	**Method:** Case-control study **Age**: Mean = 24 **Gender:** Transgender **Sexual orientation**: Not specified **Sample size:***N* = 71	Compared to the control group, transgender participants had significantly lower quality of life in the dimensions of physical and social functioning, role limitations, and vitality. In male-to-female: significant association between hormone therapy and subscales of the physical role limitations, emotional health, and social functioning; and significant association between surgical intervention and subscales of physical functioning, vitality, emotional health, and social functioning In female-to-male: significant association between hormone therapy and with subscales of vitality, emotional health, social functioning, and pain; and significant association between surgical intervention and the subscales of physical role limitations, vitality, emotional health, social functioning, and pain.
5 Simbar (2018); Iran	Explore body image and quality of life of individuals with gender dysphoria (GD) who were undergoing different treatments versus no treatments.	**Method:** Descriptive-comparative study **Age**: Range:18–45 **Gender:** Male-to-female or female-to-male transgender participants. **Sexual orientation:** Not specified **Sample size:***N* = 90	The present study demonstrated a significant higher score of quality of life and body image of GD individuals who were treated with gender-reassignment surgery in comparison with the group receiving hormone therapy and with the group who received no treatment.
6 Mohammadi (2018); Iran	Explore the life experiences of transgender persons in Iran	**Design:** Qualitative **Age**: Range: 20–40 **Gender:** Transgender **Sexual orientation: N**ot specified **Sample size:***N* = 18	Themes identified: Loss of self-confidence; Loss of legal-self; Loss of social-esteem.
7 Khorashad (2019); Iran	Evaluate the ambivalent sexism among Iranian individuals with gender dysphoria with or without disorders of sex development	**Design:** Quantitative cross-sectional **Age**: Mean = 25 **Gender:** Transgender, cisgender, and people with disorders of sex development **Sexual orientation:** Attracted to women **Sample size:***N* = 387	Scores of transgender people and people with disorders of sex development were not significantly different from each other on hostile sexism; but both groups were significantly more sexist than controls. In benevolent sexism, individuals with disorders of sex development were significantly most sexist, followed by transgender people and controls showing the least degree of sexism.
8 Roshan (2019); Iran	Report and compare the pre-school activities recalled by Iranian adults with gender dysphoria to their cisgender friends and relatives.	**Design**: Quantitative cross-sectional **Age**: Mean = 25 **Gender**: Transgender men and women. Controls: cisgender women and men. **Sexual orientation**: Not specified **Sample size:***N* = 175	Scores of recalled activities in transmen were significantly more masculine than their sex-matched cisgender women but did not differ significantly from their gender matched cisgender men. Transwomen recalled significantly more feminine play behaviors and preferences in their childhood compared to both sex matched cisgender men and gender matched cisgender women.
9 Naeimi (2019); Iran	Compare quality of life in female-to-male (female-to-male) gender identity disorder patients before and after gender reassignment surgery.	**Design:** Prospective study; quasi experimental **Age**: Mean = 34 **Gender**: Transgender (female-to-male) **Sexual orientation:** Not specified **Sample size:***N* = 42	Total mean score of quality of life significantly improved, 6 months after surgery and also in all domains: physical function, social functioning, physical problem, mental health, energy and vitality, bodily pain, and general perception of health.
10 Saeidzadeh (2019); Iran	Examine how transmen who undergo or plan to undergo medical transition construe their masculinity	**Design:** Qualitative **Age**: Not specified **Gender:** Transgender men **Sexual orientation: N**ot specified **Sample size:***N* = 14	Themes identified: Manliness; Realness; Psychological wellness.
11 Mofradidoost (2020); Iran	Compare body image concern and gender identities between post-operative transgender and cisgender persons.	**Design:** Quantitative cross-sectional, case control **Age:** No age restrictions **Gender:** Transgender men/women and cisgender men/women **Sexual orientation:** Not specified **Sample size:***N* = 96	The mean body image score in transgender participants was significantly higher than cisgender participants. Among the four gender groups, transwomen had the highest score and cismen had the lowest score.
12 Khoury (2020); Lebanon	Evaluate the adequacy of the proposed International Classification of Diseases version 11 diagnostic guidelines for Gender Incongruence of Adolescence and Adulthood in transgender people	**Design:** Cross-sectional quantitative **Age:** Mean = 30 **Gender:** Transgender **Sexual orientation:** Not specified **Sample size:***N* = 28	All the participants reported experiencing the desire to be a different gender during the index period from adolescence to adulthood. Almost all respondents reported discomfort with aspects of their body during the interview and all made behavioral changes to make themselves resemble the desired gender.
13 Collumbien (2009); Pakistan	Distinguish between three distinct groups of male and transgender sex workers in Pakistan.	**Design:** Qualitative study **Age:** Not specified **Gender:** Cisgender male and transgender participants **Sexual orientation:** Have sex with men **Sample Size:***N* = 23	Within the epidemiological category of biological males who sell sex, there are three sociologically different sexual identities: khusras (transgender), khotkis (feminized males) and banthas (mainstream male identity). While banthas do not have a stigmatized sexual identity, for khotkis and khusras the sexual identity is both a cause for discrimination as well as central to social organization and access to peer support structures. Hiding both sex work and a stigmatized identity from family, neighbors and authorities is a daily preoccupation.
14 Rehan (2011); Pakistan	Report on the genital examination of hijras.	**Design**: Quantitative cross-sectional **Age:** Mean = 25; range:13–50 **Gender:** Male-to-female transgender participants **Sexual orientation:** Not specified **Sample size:***N* = 400	The penis and testes were absent in only three hijras (0.8%). Out of remaining 397 hijras, 98.3% were circumcised and seven (1.7%) were non-circumcised.
15 Alizai (2017); Pakistan	Examine the life course of Hijras and their access to fundamental human rights	**Design:** Qualitative design **Age:** Older than 15 **Gender:** Transgender **Sexual orientation:** Not specified **Sample Size:***N* **=** 50	Themes identified: Family; School; Becoming a chela (disciple); Workplace; Authorities; Elderhood.
16 Saeed (2017); Pakistan	Investigate the complexities of the disclosure decision faced by transgender employees in a developing country across both work and non-work domains.	**Design**: Qualitative study **Age**: Range: 22–50 **Gender**: Transgender **Sexual orientation:** Not specified **Sample size:***N* **=** 16	Ten participants fully concealed their identity, four partially revealed, and the remaining two fully revealed. Disclosure was influenced by the complexities of family honor, tightly integrated family network, social obligation to get married, and prevalent religious beliefs in the society.
17 Irshad (2020); Pakistan	Describe the case of one transman from a low-income Pakistani household with regard to gender dysphoria, depression, and sex reassignment surgery.	**Design:** Case study **Age**: 25-year-old **Gender:** Transgender man **Sexual orientation: A**ttracted to women **Sample size:** N = 1	Experiences of: depression, low mood, suicidal ideation; family conflict; disapproval of mother and sisters and approval of two brothers of sex-reassignment surgery. Participant was diagnosed with gender dysphoria with comorbid depression on medications. Participant was not able to follow-up after 6 months to pursue his gender reassignment surgery.
**Violence and Discrimination**			
**First Author (Year); Country**	**Study Aims**	**Study Design and Sample Characteristics**	**Relevant Findings**
1 El Khoury (2019); Lebanon	Examine the prevalence of childhood and post-childhood experiences of sexual violence in men who have sex with men.	**Design:** Quantitative cross sectional: (secondary data analysis) **Age**: Range: 18–29 **Gender:** Cisgender male **Sexual orientation:** Homosexual (men who have sex with men) **Sample size:***N* **=** 226	Sexual violence: 48.7% experienced being forced or pressured to have sex during their lifetime. Childhood sexual abuse was correlated with more types of sexuality-related discrimination in the past year and with ever been in an abusive relationship, compared with their peers who did not report being sexually abused as a child. Sexual abuse post-childhood was correlated with weekly use of marijuana, a greater number of substances used in the past 3 months, greater number of types of sexuality-related discrimination in the past year, having been in an abusive relationship, and reporting a greater number of male sex partners in the past 3 months, in comparison with those who did not experience post-childhood sexual violence.
2 Orr (2019); Lebanon	Characterize experiences of discrimination and violence of men who have sex with men in Lebanon, with special focus on the intersection between sexual behavior and foreign-born status.	**Design:** Quantitative cross-sectional **Age**: No restrictions were reported **Gender**: Cisgender male **Sexual orientation**: Men who have sex with men. **Sample size:***N* = 292	Experiences of violence and discrimination: 42.1% experienced discrimination or violence in one or more domains over the past 12 months. Prevalence: Verbal insults (38%), physical assault (14.4%), rape (13%). Risk of experiencing any of the eight types of discrimination and violence is about twice as large for participants born outside of Lebanon than for participants born in Lebanon.
3 Mayhew (2009); Pakistan	Investigate the nature and extent of human rights abuses against three vulnerable groups (injecting drug users and male and transgender sex workers).	**Design:** Mixed methods **Age:** No specific restriction **Gender:** Cisgender men and women, and transgender women **Sexual orientation:** Not specified **Sample size:***N* = 1541	**Abuse:** All groups experienced abuse from clients and people in their neighborhoods. Among transgender and male sex workers, abuse increases the more feminized the person was. Abuse ranged from verbal abuse to physical beatings, sexual assault and rape. Qualitative data: all sex worker groups were exploited by police to negotiate bribes of money or free sex from the sex workers and their managers.
4 de Lind van Wijngaarden (2013); Pakistan	Describe the experiences of Hijras in Pakistan who face high levels of stigma, violence, and sexual abuse.	**Design:** Qualitative research **Age:** Mean = 17; range: 14–20 **Gender:** Male and transgender **Sexual orientation:** Not specified **Sample Size:***N* **=** 10	Sexual abuse: All participants reported to having been raped several times (at age of 12 years on average). Support, Stigma and discrimination: Social support needs and stigma are major problems – boys are driven out of their families and schools.
5 de Lind van Wijngaarden (2014); Pakistan	Explore the social background, sexual initiation and abuse, and subsequent sexual lives of men who have sex with men truck cleaners.	**Design:** Qualitative design **Age:** Range: 15–26 years **Gender:** Male **Sexual orientation:** Men who have sex with men **Sample Size:***N* **=** 11	The participants were raped and sexually abused during their early childhood while working as truck cleaners/helpers. Many of the participants expressed a need for rape and sexual abuse counselling – feeling ashamed, a threat to masculinity, and taboo of discussing being raped. This abuse tainted their childhood experiences and their identity as children.
6 Sadiq (2015); Pakistan	Investigate the relationship of discrimination, depression, and belief in just world among transgender (hijras) people in Pakistan	**Design:** Quantitative cross-sectional **Age**: Mean = 37 **Gender:** Transgender **Sexual orientation:** Not specified **Sample size:***N* = 153	Depression was significantly correlated with Day to Day Life Perceived Discrimination Measure, Personal and General Belief in Just World Scales. Discrimination was a significant predictor of depression among transgender people. Personal Belief in Just World was a significant moderating variable between perceived discrimination and depression.
7 Shah (2018); Pakistan	Investigate the association of social exclusion/victimization with high-risk behaviors among the transgender community in Pakistan.	**Method:** Quantitative cross-sectional **Age**: Mean = 29 **Gender:** Transgender **Sexual orientation**: Homosexual and heterosexual **Sample size:***N* = 189	Discriminatory victimization was associated with an increased risk of substance abuse and selling sex. Compared to no risk, institutional discrimination was associated with increased risk of suicidal ideation (only/total) and attempts. Selling sex and begging are also significantly associated with institutional discrimination. Forced sex at young age was significantly associated with future habit of selling sex.
Experiences with Healthcare System			
**First Author (Year); Country**	**Study Aims**	**Study Design and Sample Characteristics**	**Relevant Findings**
1 Jazi (2015); Iran	Assess the attitude of transsexual patients towards doctors’ empathy	**Design:** Quantitative cross-sectional **Age:** Mean = 24 **Gender:** Transsexual men and women **Sexual orientation:** Not specified **Sample Size:***N* = 40	Overall Empathy score: 19 (range score of 5–25). Higher levels of empathy were reported for psychiatrists in comparison to other physicians. Psychiatrists were reported to have more experience with transsexual patients which means that they were more empathetic – study showed that trained providers can provide better care.
2 Abboud (2020); Lebanon	To examine the experiences of lesbian, gay, bisexual, and transgender individuals with the healthcare system in Lebanon, and to explore their expectations from lesbian, gay, bisexual, and transgender-affirming healthcare providers	**Design:** Secondary analysis of qualitative cross-sectional data **Age:** Range: 21–28 **Gender:** Cisgender and transgender participants **Sexual orientation**: Gay/lesbian, bisexual, heterosexual **Sample size**: *N* = 13	Anticipation of discrimination (Fear of being discriminated against that leads to delaying or refusing to seek healthcare; fear of homophobia, transphobia, being outed, and constantly hearing negative comments, while navigating masculine environments). Negative experiences with healthcare providers (e.g. disclosure of sexual orientation). Negative experiences with healthcare systems (confidentiality, lack of access, financial challenges, antidiscrimination policies, geographic distance). Transgender-specific experiences: unique challenges, stigma, and discrimination, especially for transgender refugee women.

Studies are listed chronologically in alphabetical order of countries.

### 3.4 Sexual minority participants

In only one study, researchers investigated the sexual health of cisgender sexual minority women (Gereige et al., 2018). In this study, sexual minority women were compared to heterosexual cisgender women and were found to be significantly younger at sexual debut than their heterosexual counterparts (19 versus 21 years). Sexual minority women reported a significantly higher number of lifetime sexual partners (mean *n* = 13 versus *n* = 7) and experiences of unwanted sexual contact (50% versus 23%) than did heterosexual women. They were also more likely than heterosexual women to have been tested for sexually transmitted infections (22% versus 4%).

In the other studies with sexual minorities, researchers focused on sexual health among sexual minority men. Like sexual minority women, most sexual minority men had their first sexual encounter before age 18 (Mirzazadeh et al., 2014; Valadez et al., 2013; Wagner et al., 2012), and more than 10% (13.8%) reported forced sexual debut (Valadez et al., 2013). The number of sexual partners was high in most studies that assessed this outcome. In Lebanon, 78% of 2238 study participants reported more than one sexual partner (Assi et al., 2019). Other studies reported a mean of 3.9 (SD [Standard Deviation] = 5.4) (Wagner et al., 2020) and 4.8 (SD = 8.7) sex partners (Wagner et al., 2014) in the past three months, and researchers in one study of 101 participants reported that 65% had five or more sexual partners in the past year (Kassak et al., 2011). Similarly, in Egypt, the majority of sexual minority men engaged in penetrative and receptive anal sex (66%) and with more than one partner (77%); slightly over one-half (53%) reported more than three sexual partners per week (El-Sayyed et al., 2008). Most studies in Pakistan focused on sexual minority men sex workers and not surprisingly, the number of sex partners was high, ranging from 4/week to 13/month (Bokhari et al., 2007; Mir et al., 2013; Saleem et al., 2008).

#### 3.4.1 HIV rates and testing

HIV rates among sexual minority men ranged between 0% to 12% in studies conducted in six countries (Assi et al., 2019; Bokhari et al., 2007; Heimer et al., 2017; Johnston et al., 2013; Khanani et al., 2010; Maatouk, 2016; Maatouk and Abdo, 2016; Maatouk et al., 2020; Mahfoud et al., 2010; Melesse et al., 2016; Mirzazadeh et al., 2014; Mor et al., 2016; Shaw et al., 2011; Tohme et al., 2016; Valadez et al., 2013; Wagner et al., 2020; Wagner et al., 2014; Wagner et al., 2015; Zuckerman et al., 2019). In two studies, researchers estimated patterns of HIV prevalence among sexual minority men sex workers in Pakistan. In one study, the projected prevalence was 17%−22% by 2015 (Reza et al., 2013); in the other study, the projected prevalence was 24% by 2020 (Melesse et al., 2018). HIV testing (operationalized as lifetime, past-year, or past 6-month testing) ranged from 1% to 82% (Alkaiyat et al., 2014; Collumbien et al., 2008; Ghanem et al., 2020; Hawkes et al., 2009; Maatouk, 2016; Mahfoud et al., 2010; Matarelli, 2013; Mirzazadeh et al., 2014; Tohme et al., 2016; Valadez et al., 2013; Wagner et al., 2020; Wagner et al., 2014; Wagner et al., 2015; Zamani et al., 2010), with the highest rates in Lebanon (up to 82%) and the lowest rates in Pakistan (1%−7%). Two studies in Lebanon were specific to refugee sexual minority men (Syrians, Palestinians, and Iraqis), and researchers reported HIV rates at 3% and ever been tested for HIV at 48% (Tohme et al., 2016a; Tohme et al., 2016b). Among refugees, relationship status, sex work, self-identifying as gay, and fewer years living in Lebanon were predictors of HIV testing (Tohme et al., 2016b). In one study, researchers compared sexual risk behaviors between drug-using sexual minority and heterosexual men and found comparable rates of HIV testing between the two groups (40% each) (Zamani et al., 2010). Stigma, fear of being judged, negative interactions with healthcare workers, and concerns about confidentiality were among the main barriers for HIV testing reported in the studies (Alkaiyat et al., 2014; Wagner et al., 2012).

#### 3.4.2 Sexually transmitted infections (other than HIV)

Rates of human papilloma virus (9%−41%) (Assi et al., 2019; Maatouk, 2016; Maatouk et al., 2020; Tohme et al., 2016; Wagner et al., 2014), gonorrhea (17%−27%) (Assi et al., 2019; Maatouk, 2016; Maatouk et al., 2020; Wagner et al., 2014), chlamydia (17%−26%) (Assi et al., 2019; Maatouk, 2016; Maatouk et al., 2020; Wagner et al., 2014), and syphilis (3%−43%) (Assi et al., 2019; Maatouk, 2016; Maatouk et al., 2020; Wagner et al., 2014) varied greatly by country and study. Rates of hepatitis B ranged between 3%−6%, and rates of hepatitis C ranged between 7%−17% (Khanani et al., 2010; Valadez et al., 2013). In another study, researchers reported high rates of HIV and syphilis co-infection in a sample of 669 sexual minority men (32%−56%) (Johnston et al., 2013).

#### 3.4.3 HIV, sexually transmitted infections, and condom knowledge

Knowledge regarding HIV, sexually transmitted infections, and condom use was measured differently across studies, and results were mixed. In Libya, Yemen, and Jordan, knowledge about HIV and sexually transmitted infection transmission was low (17%, 28%, and 32% respectively) (Alkaiyat et al., 2014; Mirzazadeh et al., 2014; Valadez et al., 2013). For example, in the study conducted in Libya, only 1% of sexual minority men correctly identified two common sexually transmitted infection symptoms, and only 12% knew how to use condoms correctly (Valadez et al., 2013). In Pakistan, HIV, sexually transmitted infection, and condom protection knowledge was better and ranged from 36% to 70% (Saleem et al., 2008; Shaw et al., 2011). In a study of sexual minority men in Egypt, HIV and sexually transmitted infection knowledge increased with educational level (El-Sayyed et al., 2008).

#### 3.4.4 Condom use

Condom use (measured as lifetime, past six months, or last sexual encounter) varied greatly in Lebanon (46% to 70%) (Assi et al., 2019; Ghanem et al., 2020; Heimer et al., 2017; Kassak et al., 2011; Maatouk, 2016; Mahfoud et al., 2010; Wagner et al., 2012; Wagner et al., 2020; Wagner et al., 2014; Wagner et al., 2015) but was higher than in other countries such as Morocco (37% – 49%) (Johnston et al., 2013), Pakistan (3% – 35%) (Bokhari et al., 2007; Collumbien et al., 2008; Hawkes et al., 2009; Melesse et al., 2016; Saleem et al., 2008; Shaw et al., 2011), Libya (21%) (Valadez et al., 2013), Yemen (20%) (Mirzazadeh et al., 2014), Egypt (17% – 19%) (Elmahy, 2018; El-Sayyed et al., 2008), and Jordan (10%) (Alkaiyat et al., 2014). In Lebanon, sexual minority men refugees (Tohme et al., 2016) and male sex workers (who were mostly migrants from Syria and Iraq) reported using condoms inconsistently, whereas male escorts (of Lebanese nationality) reported consistent condom use (Aunon et al., 2015). In other studies, researchers suggested that condom use was higher among male sex workers whose clients included injection drug users (Collumbien et al., 2008; Shaw et al., 2011).

In some studies, condom use was positively associated with older age and negatively associated with religiosity (Elmahy, 2018; El-Sayyed et al., 2008). Participants who reported condom use also reported feeling more worried about HIV and sexually transmitted infections than those who did not report condom use (Wagner et al., 2012). Some of the main reasons for low condom use were stigma, knowing one's partner, and not practicing anal intercourse (Alkaiyat et al., 2014). Condomless anal sex was associated with greater knowledge of HIV risk, greater perceived judgmentalism in communication about sex, greater number of types of gay-related discrimination experiences, and lower general social support (Ghanem et al., 2020; Wagner et al., 2020).

#### 3.4.5 Other sexual risk behaviors

In one study (Morocco), around 65% of participants received money for sex, and 83% reported also having mostly unprotected sex with women (Johnston et al., 2013). A small percentage of male sex workers (1%) had overlapping sexual risk behaviors, defined as having sex with people who inject drugs and sharing needles (Melesse et al., 2018). Khanani and colleagues reported bridging of HIV transmission to spouses and children from sexual minority men and injection drug users through needle sharing (Khanani et al., 2011). Sexual minority men reported significantly higher rates of sharing needles or having had more than five sexual partners in their lifetime than heterosexual injection drug users (Zamani et al., 2010). In a comparative study between Arab and Jewish sexual minority men, Arabs had their first sexual encounter at a younger age, had a greater number of sexual partners, and were more likely to pay for sex and perform unprotected anal intercourse. However, Arab sexual minority men were less likely to perform receptive anal intercourse and were less likely to engage in group sex than Jewish sexual minority men (Mor et al., 2016).

#### 3.4.6 Pre-exposure prophylaxis use

In the only study where authors investigated factors associated with willingness to take pre-exposure prophylaxis, 72% of young sexual minority men reported that it was very/somewhat likely that they would use pre-exposure prophylaxis. Knowledge of HIV risk, awareness of pre-exposure prophylaxis, having had recent condomless anal sex with partners whose HIV status was positive or unknown, and use of substances just prior to or during sex were all positively correlated with greater willingness to use pre-exposure prophylaxis (Storholm et al., 2019).

### 3.5 Gender minority participants

All studies with gender minority participants (transgender, transsexual, and hijras) were conducted in Pakistan, Lebanon, or Iran. Among transgender samples, the mean age of first sexual encounter ranged from 14 years to 16 years (Hawkes et al., 2009; Moayedi-Nia et al., 2019; Saleem et al., 2008), and 18% reported forced first sex (Hawkes et al., 2009). As noted earlier, the language used in some studies is not consistent with language used in most Western studies and, in some cases, may also seem outdated, incorrect, or imprecise. For example, Akhtar and colleagues (Akhtar et al., 2012) referred to their participants as transgender men when describing transgender women. Participants were described as “transgender men (males by nature but appearing as women); including a few individuals whom [*sic*] had undergone physical transformations through sex reassignment surgery, use of hormones or silicone fillings (p.1).”

#### 3.5.1 HIV rates and testing

HIV rates were similar between transgender sex workers (1% – 22%) (Altaf et al., 2012; Bokhari et al., 2007; Hawkes et al., 2009; Khan et al., 2008; Melesse et al., 2016; Reza et al., 2013) and transgender individuals (2% – 22%) (Akhtar et al., 2012; Kaplan et al., 2016; Moayedi-Nia et al., 2019). Among transgender sex workers living with HIV, 17% had been diagnosed recently with HIV, whereas 83% had chronic HIV infections (Hasan et al., 2018). Although a diagnosis of HIV added to the social stigmatization of transgender sex workers, many clients continued to have sex with transgender sex workers despite knowledge of their HIV status. Transgender sex workers reported experiencing humiliation from their fellow transgender sex workers after their HIV diagnosis, which forced them to be socially isolated (Usman et al., 2018). HIV testing ranged from 7% to 14% among Pakistani transgender sex workers (Altaf et al., 2012; Collumbien et al., 2008; Hawkes et al., 2009) to 43% among transgender women in Lebanon (Kaplan et al., 2016). In two studies, researchers adapted and tested an HIV prevention intervention, TransAction (Baynetna), to assess feasibility and acceptability among transgender women in Lebanon. Participants in these studies evaluated the intervention to be feasible and acceptable, and HIV testing increased among study participants (Kaplan et al., 2020; Kaplan et al., 2019).

#### 3.5.2 HIV, sexually transmitted infections, and condom knowledge

Similar to research among cisgender sexual minority men, knowledge regarding HIV, sexually transmitted infections, and condom use was measured differently across studies, and results were mixed. Overall good HIV and sexually transmitted infection knowledge (transmission, prevention, condom use, treatment) ranged from 31% to 78% across studies (Hawkes et al., 2009; Khan et al., 2008; Saleem et al., 2008; Shaw et al., 2011). For example, in one study, most participants (68%) had heard of HIV and associated HIV risk reduction with condom use (69%), avoidance of anal sex (73%), or needle sharing (87%) (Khan et al., 2008).

#### 3.5.3 Condom use

Condom use was low and ranged from 4% to 45% for last anal intercourse (Altaf et al., 2012; Bokhari et al., 2007; Collumbien et al., 2008; Hawkes et al., 2009; Kaplan et al., 2016; Khalid and Martin, 2019; Khan et al., 2008; Melesse et al., 2016; Moayedi-Nia et al., 2019; Saleem et al., 2008; Shaw et al., 2011).

#### 3.5.4 Other sexual risk behaviors

In one study, 3% of transgender sex workers reported having unprotected sex with people who injected drugs and shared needles (Melesse et al., 2018). Since most studies in Pakistan involved transgender sex workers, the number of sex partners was high and ranged from 4 partners/week to 18 partners/month (Altaf et al., 2012; Bokhari et al., 2007; Saleem et al., 2008).

Transgender women described multiple factors related to sexual risk behaviors, including lack of social, emotional, physical, and financial safety; they also reported coercive sex and inability to consistently use condoms because of stigma and fear of being judged (Kaplan et al., 2015). They described using high-risk and unprotected sexual relationships as a way to be accepted aswomen, to make money, for protection, and to obtain shelter. In evaluating the risks of unprotected sex, participants noted that the risk of HIV was less important than their other problems, such as depression and hopelessness (Eftekhar et al., 2020). Notably, there were no studies of pre-exposure prophylaxis use or knowledge among transgender people, despite their engagement in high-risk behaviors.

### 3.6 Mental health

In 20 of the studies reviewed, researchers focused on mental health. These studies were conducted in Iran (*n* = 8), Lebanon (*n* = 6), Pakistan (*n* = 5), and Kuwait (*n* = 1). We first describe the studies conducted with sexual minority participants (*n* = 7), followed by studies conducted with gender minority participants (*n* = 13).

### 3.7 Sexual minority participants

#### 3.7.1 Depression, anxiety, and suicidal thoughts and behaviors

Compared to heterosexuals, sexual minority individuals reported higher levels of anxiety (Nematy and Oloomi, 2016), social isolation, feelings of defectiveness/shame, and emotional inhibition (Nematy et al., 2014). There was no significant difference in attachment styles (defined as closeness, dependency, and anxiety) between bisexual/lesbian women and gay men (Nematy and Oloomi, 2016). It is notable that this was the only study in which researchers reported data on mental health among sexual minority women. Among sexual minority men, depressed mood was reported by 41% of the sample, with 26% having symptoms consistent with a clinical diagnosis of depression and 16% having major depression (Wagner et al., 2019). One third of sexual minority men (33%) reported suicidal thoughts, including having a plan (Wagner et al., 2019).

#### 3.7.2 Mental health risks and buffers

Sexual minority individuals whose families were not aware of their sexual identity scored significantly higher on social isolation, vulnerability to harm/illness, and emotional inhibition than those whose families were aware (Nematy et al., 2014). Although sexual minority participants generally endorsed low levels of internalized homonegativity (Michli and El, 2020), those who reported less comfort with their sexual identities reported higher levels of anxiety (Nematy and Oloomi, 2016). Sexual minority individuals also reported high levels of parental rejection (Michli and El, 2020) and significant conflicts and tensions between their sexual and religious identities (Scull and Mousa, 2017). Sexual minority individuals described risks associated with their sexual identities (such as fear of living openly or someone discovering their sexual orientation) and highlighted the need to build accepting sexual minority communities (Scull and Mousa, 2017). Sense of belonging to a community buffered the impact of internalized homonegativity (Michli and El, 2020). Although the above mentioned researchers (Michli and El, 2020; Nematy and Oloomi, 2016; Nematy et al., 2014; Scull and Mousa, 2017) included sexual minority women in their overall sample, none disaggregated the findings.

Unemployment, lack of legal resident status, discrimination experiences, discomfort with being a sexual minority, and lack of social support were significantly associated with major depression among sexual minority men (Wagner et al., 2019). Participants described experiencing multiple forms of stigma and discrimination at home and in the workplace or at school; these experiences negatively impacted self-esteem and mental health and increased internalized homophobia. Coping mechanisms included concealing sexual minority status, turning to the internet for social and sexual networking, avoiding in-person social relations, focusing on work and academic performance, stepping back from religion, and using alcohol (Wagner et al., 2013). Psychological well-being among sexual minority men was described in terms of relationships with and disclosure of sexual identity to family, friends, and co-workers. More than half of sexual minority men (61%) reported close relationships with their families that were supportive and affectionate and also reported that they were able to disclose their sexual orientation to at least one member of their family; fewer participants disclosed their sexual identities to friends or co-workers (32%) (Wagner et al., 2013). Participants described the importance of a safe space, finding other sexual minority men, balancing safety with visibility, and the struggles they faced in finding support within the sexual minority community (Mutchler et al., 2018).

### 3.8 Gender minority participants

#### 3.8.1 Depression, anxiety, and suicidal thoughts and behaviors

Depression rates ranged from 45%−66% (Ibrahim et al., 2016; Kaplan et al., 2016). Among transgender women, 29% met criteria for diagnosable mood disorders compared to 24% of transgender men. Rates of anxiety disorders were lower among transgender women (7%) than among transgender men (36%) (Aghabikloo et al., 2012). Between one-quarter to one-half (21%−55%) of participants reported suicidal thoughts. Reporting suicidal thoughts was significantly associated with depression, drug use, lower general social support, lower social integration, lower levels of support from peers, being more open about transgender identity in public, and any hormone use (Azeem et al., 2019; Ibrahim et al., 2016; Kaplan et al., 2016). Almost one-third to one-half of participants (29%−46%) reported having attempted suicide (Bin et al., 2019; Kaplan et al., 2016).

#### 3.8.2 Mental health risks and buffers

In Pakistan, transgender participants described early realization of their differences, which was accompanied by physical and psychological reprimands from their family; they also described poor living conditions, feeling lonely, and anticipating dying in isolation (Abdullah et al., 2012). Almost half of transgender participants (46%) reported a medium level of psychological resilience while 25% reported a high level; similarly, 44% of transgender participants reported a medium level of self-esteem while 26% reported a high level (Akhtar and Bilour, 2020). Transgender participants residing with their gurus had a significantly higher level of psychological resilience and self-esteem compared to those living alone or with friends (Akhtar and Bilour, 2020). In one intervention study, researchers used an empowerment model-based training to improve quality of life among transgender individuals in Iran (Asadi et al., 2020). The treatment group showed statistically greater improvement on quality of life and mental health than the control group.

### 3.9 Violence and discrimination

In seven studies (five in Pakistan and two in Lebanon), researchers investigated different forms of violence and discrimination. We first describe the studies conducted with sexual minority participants (*n* = 4), followed by the studies conducted with gender minority participants (*n* = 4). There is one overlapping study that included sexual and gender minority participants.

### 3.10 Sexual minority participants

All research on violence among sexual minority people involved sexual minority men. Rates of sexual abuse (sexual harassment, rape, forced sex) tended to be high among sexual minority men, ranging from 11%−49% (El Khoury et al., October 2019; Mayhew et al., 2009; Orr et al., 2019). In one study, 17% of the participants were younger than 13 when they first experienced sexual violence (El Khoury et al., October 2019); similarly in another study, participants described being raped and sexually abused during early childhood while working as truck cleaners/helpers (de Lind van Wijngaarden and Schunter, 2014). Being sexually abused as a child was associated with experiencing more types of sexuality-related discrimination in the past year and being in an abusive relationship (El Khoury et al., October 2019). Other forms of abuse were also high; verbal abuse ranged from 42%−56% (Mayhew et al., 2009; Orr et al., 2019) and physical abuse from 26%−44% (Mayhew et al., 2009). Participants also described multiple forms of abuse by the police that ranged from 11% to 50% (Mayhew et al., 2009). In Lebanon, foreign-born sexual minority men were twice as likely as native-born sexual minority men to report any type of discrimination or violence (Orr et al., 2019).

### 3.11 Gender minority participants

In a qualitative study conducted in Pakistan (*N* = 10), all participants reported being raped several times starting on average at 12-years-old (de Lind van Wijngaarden et al., 2013). In a study of transgender individuals in Lebanon, researchers found that 71% of participants reported experiencing violence during childhood or adolescence, including psychological (90%), physical (75%), and sexual (60%) violence (Khoury et al., 2021). Similarly in Pakistan, 28% – 62% of transgender participants reported having experienced sexual assault (Shah et al., 2018), 36% (Mayhew et al., 2009) to 78% (Shah et al., 2018) reported physical abuse, and 59% (Mayhew et al., 2009) to 91% (Shah et al., 2018) reported at least one type of institutional discrimination (law enforcement, doctors, hospitals, and housing). Physical violence, discrimination, or institutional discrimination were associated with higher risk for suicidal ideation, depression, substance abuse, or engaging in sex work (Mayhew et al., 2009; Shah et al., 2018).

### 3.12 Experiences with the healthcare system

In only two studies, researchers investigated experiences of sexual and gender minority people with the healthcare system. In Lebanon, researchers found that most sexual and gender minority participants reported anticipation of discrimination and negative and positive experiences with healthcare providers and systems. They described their expectations of affirming providers as respectful, inclusive, and knowledgeable. In this study, transgender participants, especially refugee transgender women, reported unique challenges, stigma, and discrimination (Abboud et al., 2020). Transgender participants in Iran perceived psychiatrists as being more empathetic, non-discriminatory, and providing better care to transgender participants than other healthcare providers (Jazi et al., 2015).

### 3.13 Studies about gender

In 17 studies (11 in Iran, five in Pakistan, and one in Lebanon), researchers focused on different aspects of gender among gender minority individuals.

#### 3.13.1 . Gender identity

In a comparative study between transgender individuals living in Iran and transgender individuals living in the Netherlands, Iranian participants had significantly higher rates of gender dysphoria and psychological symptoms than Dutch participants (Shirdel-Havar et al., 2019). In a study of transgender people in Lebanon, all (*N* = 28) participants reported experiencing a desire to be a different gender from adolescence to adulthood, and almost all (*n* = 27) reported discomfort with different aspects of their bodies (Khoury et al., 2021).

#### 3.13.2 . Gender roles

In three studies in Iran, researchers compared gender roles between cisgender and transgender participants (Alavi et al., 2015; Khorashad et al., 2019; Roshan et al., 2019). For example, Alavi and colleagues reported that compared to cisgender male and female participants, transgender participants had the highest and lowest score on the Gender Masculine scale, respectively; transgender men had the lowest score on the Gender Feminine scale of all groups (Alavi et al., 2015).

#### 3.13.3 Gender affirming treatments

In several studies, researchers compared transgender individuals undergoing different treatments and found that quality of life and body image scores were significantly higher among those who underwent gender-reassignment surgery than those who received only hormone therapy or who received no treatment (Fallahtafti et al., 2019; Mofradidoost and Abolghasemi, 2020; Naeimi et al., 2019; Simbar et al., 2018). Transgender men undergoing or planning to undergo medical transition described their identities in terms of manliness (manlier than cisgender men), realness (they are “real” transgender people compared to “fake” transgender women), and psychological wellness (Saeidzadeh, 2020).

#### 3.13.4 Quality of life

In one study of transgender people in Iran, a majority of participants had at least a high school diploma (77%), lived in urban areas (82%), were employed (57%), were single (93%), and reported less than six months of hormonal treatment (61%) (Hedjazi et al., 2013). Compared to the general population, quality of life of transgender participants was significantly lower in multiple dimensions including physical functioning, social functioning, role limitations, and vitality (Valashany and Janghorbani, 2018). When asked about their lived experiences related to gender, transgender individuals described loss of self-confidence, loss of legal-self, and loss of social esteem (Mohammadi, 2018). Transgender participants in Pakistan described various forms of rejection, discrimination, violence, and abuse from family members during childhood and from the general community and in the workplace during adulthood; elderly participants also reported difficult circumstances due to extreme poverty, disease, isolation, and lack of support (Alizai et al., 2017; Collumbien et al., 2009; Irshad et al., 2020). A majority (63%) of participants in a study of transgender people in Pakistan reported fully concealing their transgender identity in work and non-work domains; disclosure was influenced by the complexities of family honor, tightly integrated family networks, social obligations to get married, and national religious beliefs (Saeed et al., 2018). In addition, participants (93%) experienced social rejection from family, friends, or co-workers because of their gender identity (Khoury et al., 2021).

## 4 Discussion

Our goal in this scoping review was to comprehensively examine the literature related to the health of sexual and gender minority people in the Middle East and North Africa and to identify research gaps and priorities. Based on our review, we found that there were major gaps in the literature. An important one was the paucity of research conducted with sexual minority women; only five studies included women, and only one of these focused on sexual minority women. Researchers in the Middle East and North Africa have focused predominately on sexual minority men and transgender women. Another significant gap was the lack of focus on health issues beyond sexual behaviors and sexual health. We did not find a single paper focused on an aspect of physical health apart from sexual health.

We found the need to address the high rates of HIV and other sexually transmitted infections, low rates of both condom use and HIV/sexually transmitted infection testing, and lack of appropriate knowledge related to HIV and sexually transmitted infection prevention and treatment, including pre-exposure prophylaxis. In only one study, researchers addressed pre-exposure prophylaxis intake among sexual minority men and found high willingness to take it (Storholm et al., 2019); however, other factors associated with pre-exposure prophylaxis, such as cost, availability, and access, need to be investigated among sexual and gender minority people who are at high risk for HIV.

Mental health concerns, including depression, anxiety, and suicidal thoughts and behaviors, appear to be prevalent among sexual and gender minority people in the Middle East and North Africa. Although family and community support was highlighted as a buffer, family rejection, conflicts between sexual identity and religion, and challenges in finding community were common issues and concerns. Sexual and gender minority people also reported high rates of discrimination and alarming rates of violence, particularly among the most vulnerable groups, such as youth, gender nonconforming people, and refugees. We found only one intervention study in which researchers aimed at improving mental health, which, although successful, included only transgender people in Iran (Asadi et al., 2020). In another intervention study (among transgender people in Lebanon), researchers focused on improving sexual health outcomes but also showed improvement in mental health outcomes (Kaplan et al., 2020; Kaplan et al., 2019). Missing from the literature are larger-scale studies among sexual and gender minority people in the region to provide more precise information about rates of mental health concerns, as well as risk and protective factors associated with such concerns. Research on the mental health of sexual minority women and studies with comparison of samples based on gender and sexual identity are greatly needed.

In general, sexual and gender minority people access healthcare at lower rates than cisgender heterosexual people (Kates et al., 2018; Macapagal et al., 2016; Ward et al., 2014). The reasons are multifactorial. However, lack of culturally competent care is a large barrier to accessing healthcare (Brummett and Campo-Engelstein, 2021; Göçmen and Yılmaz, 2017; Kattari et al., 2021). Access to gender affirming treatments is associated with improved quality of life, amplifying the need for affirming and competent healthcare for gender minority people globally, but perhaps particularly in the Middle East and North Africa. It is noteworthy that in only two studies, researchers examined healthcare experiences. Understanding the unique experiences of sexual and gender minority people in the region has important implications for educational and curricular reform and training of current and future healthcare providers. Based on existing research, sexual and gender minority-specific training of healthcare providers can increase knowledge and awareness of sexual and gender minority health issues and contribute to affirming and inclusive care (Elertson and McNiel, 2021; Fauer et al., 2020; Gibson et al., 2020).

### 4.1 Gaps in country representation

All except 10 of the studies reviewed were conducted in Lebanon, Pakistan, or Iran, which may provide a highly skewed picture of sexual and gender minority health in the region. As noted earlier, same-sex relationships and gender non-conformity are criminalized in almost all countries in the Middle East and North Africa. These laws make it nearly impossible for advocacy organizations to work on issues related to sexual orientation and gender identity and make it challenging for researchers to conduct research with sexual and gender minority people (Human Rights Watch 2018). Nearly all (20 of 23) studies conducted in Iran focused on transgender individuals. This may be because Iran is one of two countries in the region to provide transgender individuals the conditional right to have their identity recognized by the law (Being Transgender in Iran 2016). However, the conditions for actualizing that right (psychiatric diagnosis of gender identity disorder and completion of gender affirming surgery) reinforce the belief that transgender people are psychologically and sexually unfit and require treatment to become “normal.” Similarly, there were also a large number of studies (28 of 32) in Pakistan conducted with transgender individuals. In 2018, the Pakistani parliament passed the “Transgender Persons (Protection of Rights) Act” which established broad protections for transgender people. As demonstrated in this review, despite the implementation of these laws in Iran and Pakistan, transgender people continue to face stigma and discrimination based on their gender identity.

Most of the research conducted in Lebanon was among sexual minority men (23 of 33 studies). Despite social, religious, and legal conservativism, Lebanon has witnessed growing advocacy, research, and visibility of sexual minority people relative to other countries in the region (Moussawi, 2017). This has allowed researchers to conduct studies, many in collaboration with sexual and gender minority-friendly community-based organizations (for example, see Kaplan and colleagues’ study with transgender women) (Kaplan et al., 2020).

Based on the identified research gaps, recommendations include expanding research beyond Lebanon, Pakistan, and Iran to represent more countries in the Middle East and North Africa. This could include collaborations between researchers and community-based organizations from different countries and exchanging lessons learned in the areas of sexual and gender minority health research.

### 4.2 Gaps in methodological designs

Most of the studies used cross-sectional quantitative designs, and only two studies tested an intervention (Asadi et al., 2020; Kaplan et al., 2020; Kaplan et al., 2019). This highlights the need for additional qualitative or mixed-methods research to better understand the complexities of the experiences of sexual and gender minority individuals; there is also a need for longitudinal studies to understand the impact of societal and legal changes on sexual and gender minority health over time. Most studies used non-probability sampling, and only five studies used stratified random selection. This type of sampling is used widely with hard-to-reach populations and can provide valuable information; however, non-probability sampling methods are subject to selection bias, and it is not possible to know the extent to which the findings accurately represent and characterize sexual and gender minority populations. Although challenging, researchers should advocate that measures of sexual orientation and gender identity be included in country-level surveys, especially nationally representative studies.

Development and implementation of multi-level interventions are needed to prevent and address the adverse sexual and gender minority health outcomes. In the two intervention studies (Asadi et al., 2020; Kaplan et al., 2020; Kaplan et al., 2019) included in this review, researchers showed promising improvements in quality of life and in the emotional, mental, and sexual health of transgender individuals. Given the challenges in conducting this type of research in the region, we highly encourage researchers to employ participatory approaches that involve multiple stakeholders from the community to develop and implement interventions, ensure trust and representation of sexual and gender minority individuals, and establish longstanding relationships (Northridge et al., 2007; Wright et al., 2017).

### 4.3 Gaps in representation and measurement of sexual and gender minority identities

Although the number of studies with sexual and gender minority individuals is growing in the region (11 studies between 2000 and 2010 compared with 87 studies between 2011 and 2021), only one exclusively focused on cisgender sexual minority women (Gereige et al., 2018). Researchers in five studies included sexual minority women in their overall sample, but none disaggregated the findings (Abboud et al., 2020; Michli and El, 2020; Nematy and Oloomi, 2016; Nematy et al., 2014; Scull and Mousa, 2017). Only 10 studies included transgender men. This highlights the need for more inclusive research of the multiple sexual and gender minority identities, particularly cisgender sexual minority women and transgender men. We also recommend better measurement of sexual orientation. Although sexual orientation consists of at least three overlapping but distinct dimensions (identity, attraction, and behavior), most researchers assessed only one dimension—mostly sexual behavior (e.g., men who have sex with men).

Another limitation is the conflation of sexual minority men and transgender women as sexual risk categories in multiple studies. Future researchers needs to acknowledge that sexual health risks and protective factors impact sexual minority men and transgender women differently and that these identities should be seen as having unique risks and resiliencies (Kaplan et al., 2016; Perez-Brumer et al., 2016; Poteat et al., 2016).

Several studies used outdated terminology when describing gender minority individuals. In a few studies conducted in Pakistan with transgender women, researchers referred to their participants as transgender men in reference to their assigned (male) sex at birth (see for example, Akhtar and colleagues) (Akhtar et al., 2012). In Iran, some researchers investigated personality disorders among transgender participants and referred to their participants’ early questioning of their gender identity as early onset of “symptoms.” (Meybodi et al., 2014; Shirdel Havar et al., 2015) As mentioned earlier, this type of research or terminology reinforces stigma and stereotypes about transgender people and contributes to these individuals’ emotional and psychological concerns.

### 4.4 Gaps in measuring different health outcomes

Although researchers assessed multiple health outcomes in many studies, more than half of the studies (53%) focused on sexual health. However, none of the studies addressed positive aspects of sexual health, such as pleasure, communication, or healthy relationships. In addition, none of the studies focused on physical health outcomes, such as cancer, diabetes, or cardiovascular issues. Although in several studies researchers assessed health behaviors, such as smoking or alcohol and other drug use, no study had as a primary objective understanding antecedents or consequences of these health behaviors. In very few studies, researchers assessed protective factors, such as coping, family acceptance, social support, and community belonging. More research is needed to understand the role of these protective factors—information that is essential in the development of prevention and intervention strategies.

We found a few studies where researchers focused on individual-level minority stress (e.g., as identity concealment, internalized homonegativity), but none of the researchers focused on the impact of structural-level minority stressors on sexual and gender minority health. In the Middle East and North Africa, where anti-sexual and gender minority laws and norms are widespread, identifying appropriate ways to assess structural stigma and its health consequences is critically important in the development of interventions that mitigate the impact of structural stigma.

### 4.5 Limitations of this scoping review

To our knowledge, this was the first review where the aim is to systematically examine research on sexual and gender minority health in the Middle East and North Africa. We used a systematic search strategy that can be replicated, which will facilitate similar future reviews. However, there are some limitations that should be considered in evaluating our findings. First, we limited our review primarily to studies published in English. Six studies in other languages were excluded, and these may have contained relevant information. It is also possible that searches of four databases did not identify all eligible articles. We excluded gray literature such as dissertations, guidelines, and reports. In recent years, there has been an increasing number of community-based organizations conducting research with sexual and gender minority people in the Middle East and North Africa. For example, the Heartland Alliance International published in an on-line report the needs, vulnerabilities, and experiences of sexual and gender minority Syrian refugees in Lebanon (Heartland Alliance International 2014). It should also be noted that none of the authors currently lives in the Middle East and North Africa. Although two authors were born and raised in Lebanon and continue to be involved in sexual and gender minority research and advocacy in Lebanon and the region, our inclusion/exclusion criteria and methods of reporting the findings through a largely Western lens may not reflect priorities of sexual and gender minority communities in the region. Finally, there isn't a single definition of the Middle East and North Africa, leading to differences regarding which countries should be included.

## 5 Conclusion

In this scoping review, we highlighted an increasing interest in sexual and gender minority health in the Middle East and North Africa, as evidenced by the growing number of research articles published, especially in the last decade. However, researchers have mostly studied sexual and gender minority health in three countries and have focused almost exclusively on sexual minority men and transgender women. The breadth of research is limited, with the majority of researchers measuring sexual health outcomes. As is the case in many other regions of the world, research on sexual and gender minority health in the Middle East and North Africa relies almost exclusively on cross-sectional designs. Based on research gaps identified in this review, recommendations include: 1) expanding research beyond Lebanon, Pakistan, and Iran to represent more countries in the region; 2) expanding research beyond sexual minority men and transgender women samples and avoiding the conflation of these identities in studies of sexual risks; 3) investigating health outcomes beyond sexual health and risk behaviors; and 4) increasing the number of studies that use longitudinal designs and that develop and test interventions. Adding questions about sexual and gender identity on national health surveys would provide critically important information that could be used to inform health policy and interventions. Nurses and other healthcare providers need to be educated about the prevalence of health disparities in this population. Nurses play a significant role in health promotion efforts and in translating research to inform nursing education and practices. Given the advancement in sexual and gender minority advocacy and the historical social and legal changes impacting sexual and gender minority people in many parts of the Middle East and North Africa, there are multiple opportunities for researchers, nurses, and other healthcare providers to contribute to improving health outcomes of sexual and gender minority people.

## Contribution of the paper

What is already known about the topic?

• Previous researchers from western countries such as those in North America and Europe have demonstrated that sexual and gender minority people experience significant health disparities.
• There are high rates of discrimination and violence, including state violence, committed against sexual and gender minority people in the Middle East and North Africa.

What this paper adds

• We found that most research conducted with sexual and gender minority people in the Middle East and North Africa is on human immunodeficiency virus (HIV) and sexual risk behaviors among sexual minority men and transgender women, with much less attention to sexual minority women and health outcomes other than HIV and sexually transmitted infections.
• Consistent with research from other parts of the world, substantial sexual and gender minority-related health disparities, such as those related to sexual and mental health, discrimination, and violence, exist among sexual and gender minority people in the Middle East and North Africa.
• Most research involving sexual and gender minority people in the Middle East and North Africa has been conducted in three countries, Lebanon (*n* = 33), Pakistan (*n* = 32), and Iran (*n* = 23); the number of studies conducted in the remaining countries ranged from 0 to 2.

## Funding sources

The authors did not receive funding for the conduct of this study.

## CRediT author statement

**Sarah Abboud:** conceptualization, methodology, formal analysis, writing- original draft preparation, reviewing, and editing, project administration; **Cindy Veldhuis:** methodology, writing- original draft preparation, reviewing, and editing; **Suha Ballout**: methodology, writing- original draft preparation, reviewing, and editing; **Fatima Nadeem:** formal analysis, investigation, data curation; **Kate Nyhan**: methodology, resources, data curation; **Tonda Hughes**: conceptualization, methodology, writing- reviewing and editing, supervision.

## Declaration of Competing Interest

The authors declare that they have no known competing financial interests or personal relationships that could have appeared to influence the work reported in this paper.

## Appendix Supplementary materials

Image, application 1

Image, application 2
