# Supplementary material for: Sexual and gender minority health in the Middle East and North Africa Region: A scoping review
Source: Int J Nurs Stud Adv. 2022 Jun 27;4:100085. doi: 10.1016/j.ijnsa.2022.100085 (PMC11080540; doi:10.1016/j.ijnsa.2022.100085)
Supplement: Supplementary file 1 [file mmc1.docx]

**Appendix A: Bibliographic Database Searches**

# **Medline**

| Ovid MEDLINE(R) ALL <1946 to February 26, 2021> | |  |
| --- | --- | --- |
|  |  |  |
| 1 | [MEDLINE All search history] | 0 |
| 2 | (gender* adj2 nonconform*).mp. | 535 |
| 3 | (gender* adj2 non-conform*).mp. | 149 |
| 4 | (trans adj (female* or male* or man or men or women or woman or boy* or girl*)).mp. | 373 |
| 5 | (trans adj (population* or patient* or participant* or subject* or adolescent* or teen* or child* or individual* or people or person* or youth*)).mp. | 375 |
| 6 | agender*.mp. | 17 |
| 7 | bicurious.mp. | 3 |
| 8 | bigender*.mp. | 6 |
| 9 | bisexual*.mp. | 10996 |
| 10 | cross sex.mp. | 533 |
| 11 | crossgender.mp. | 2 |
| 12 | DSD.mp. | 2004 |
| 13 | gay.mp. | 11865 |
| 14 | gays.mp. | 466 |
| 15 | gender change.mp. | 77 |
| 16 | gender crossing.mp. | 4 |
| 17 | gender dysphori*.mp. | 1478 |
| 18 | gender fluid*.mp. | 32 |
| 19 | gender identit*.mp. | 21014 |
| 20 | gender incongruen*.mp. | 215 |
| 21 | gender minorit*.mp. | 5087 |
| 22 | gender neutral.mp. | 469 |
| 23 | gender queer.mp. | 19 |
| 24 | gender transition*.mp. | 190 |
| 25 | gender varian*.mp. | 159 |
| 26 | genderless.mp. | 12 |
| 27 | genderqueer*.mp. | 88 |
| 28 | GLB.mp. | 422 |
| 29 | GLBQ.mp. | 3 |
| 30 | GLBs.mp. | 17 |
| 31 | GLBT.mp. | 105 |
| 32 | GLBTQ.mp. | 14 |
| 33 | heteroflexible.mp. | 3 |
| 34 | homosexual*.mp. | 36048 |
| 35 | intersex*.mp. | 3259 |
| 36 | lesbian*.mp. | 7198 |
| 37 | lesbigay*.mp. | 5 |
| 38 | LGB.mp. | 1067 |
| 39 | LGBQ.mp. | 118 |
| 40 | LGBS.mp. | 69 |
| 41 | LGBT*.mp. | 2805 |
| 42 | men who have sex with men.mp. | 12263 |
| 43 | mostly-heterosexual.mp. | 125 |
| 44 | MSM.mp. | 10989 |
| 45 | MSMW.mp. | 126 |
| 46 | nonbinary.mp. | 340 |
| 47 | non-binary.mp. | 335 |
| 48 | nonheterosexual*.mp. | 155 |
| 49 | non-heterosexual*.mp. | 286 |
| 50 | queer.mp. | 1475 |
| 51 | queers.mp. | 26 |
| 52 | same gender loving.mp. | 9 |
| 53 | same sex couple*.mp. | 366 |
| 54 | same sex relations*.mp. | 333 |
| 55 | same-sex attract*.mp. | 301 |
| 56 | sexual identit*.mp. | 1914 |
| 57 | sexual minorit*.mp. | 3022 |
| 58 | sexual orientation*.mp. | 6309 |
| 59 | sexual preference*.mp. | 650 |
| 60 | SGM.mp. | 636 |
| 61 | third gender*.mp. | 44 |
| 62 | transboy*.mp. | 7 |
| 63 | transex*.mp. | 154 |
| 64 | transfeminine.mp. | 85 |
| 65 | transgender*.mp. | 8009 |
| 66 | transgirl*.mp. | 9 |
| 67 | transm#n.mp. | 263 |
| 68 | transmasculine.mp. | 137 |
| 69 | transsex*.mp. | 4521 |
| 70 | trans-sex*.mp. | 81 |
| 71 | trans-spectrum.mp. | 6 |
| 72 | transwom#n.mp. | 239 |
| 73 | two-spirit*.mp. | 80 |
| 74 | women loving women.mp. | 8 |
| 75 | women who have sex with women.mp. | 180 |
| 76 | WSW.mp. | 156 |
| 77 | WSWM.mp. | 19 |
| 78 | or/2-77 | 83372 |
| 79 | exp "sexual and gender minorities"/ | 7787 |
| 80 | bisexuality/ | 4279 |
| 81 | transsexualism/ | 3847 |
| 82 | exp homosexuality/ | 30473 |
| 83 | gender identity/ | 18857 |
| 84 | health services for transgender persons/ | 149 |
| 85 | gender dysphoria/ | 560 |
| 86 | exp "Disorders of Sex Development"/ | 33528 |
| 87 | or/79-86 | 87127 |
| 88 | 78 or 87 | 113900 |
| 89 | [MENA region] | 0 |
| 90 | exp middle east/ or exp africa, eastern/ or exp africa,northern/ or exp pakistan/ or arabs/ | 256658 |
| 91 | (afghan* or algeria* or bahrain* or djibouti* or egypt* or iran* or iraq* or jordan* or kuwait* or lebanon or lebanese or libya* or mauritania* or morocco or morroccan* or oman* or palestin* or pakistan* or qatar* or saudi arabia* or saudi* or somalia* or sudan* or syria* or tunisia* or united arab emirates or emirati* or yemen* or middle east* or north africa* or west bank or gaza* or mena or maghreb* or israel* or arab* or druze).mp. | 408727 |
| 92 | (Kabul or Algiers or Manama or Djibouti or Cairo or Tehran or Baghdad or Amman or Kuwait City or Beirut or Tripoli or Nouakchott or Rabat or Muscat or Ramallah or Jerusalem or Islamabad or Doha or Riyadh or Mogadishu or Khartoum or Damascus or Tunis or Abu Dhabi or Sana'a or Sanaa).mp. | 28966 |
| 93 | or/90-92 | 506324 |
| 94 | [summation of LGBT and MENA] | 0 |
| 95 | 88 and 93 | 2164 |
| 96 | limit 95 to yr="2000 - 2020" | 1689 |

The Ovid Medline search can be rerun at <http://access.ovid.com/demo/ovidsptools/launcher/dlp_launcher.html>.

# **CINAHL**

Interface - EBSCOhost Research Databases
Search Screen - Advanced Search
Database - CINAHL Complete

| **#** | **Query** | **Limiters/Expanders** | **Results** |
| --- | --- | --- | --- |
| S8 | S3 AND S7 | Limiters - Published Date: 20000101-20211231  Expanders - Apply equivalent subjects  Search modes - Boolean/Phrase | 1,050 |
| S7 | S4 OR S5 OR S6 | Expanders - Apply equivalent subjects  Search modes - Boolean/Phrase | 124,059 |
| S6 | (Kabul or Algiers or Manama or Djibouti or Cairo or Tehran or Baghdad or Amman or Kuwait-City or Beirut or Tripoli or Nouakchott or Rabat or Muscat or Ramallah or Jerusalem or Islamabad or Doha or Riyadh or Mogadishu or Khartoum or Damascus or Tunis or Abu-Dhabi or Sana'a or Sanaa) | Expanders - Apply equivalent subjects  Search modes - Boolean/Phrase | 12,055 |
| S5 | (afghan* or algeria* or bahrain* or djibouti* or egypt* or iran* or iraq* or jordan* or kuwait* or lebanon or lebanese or libya* or mauritania* or morocco or morroccan* or oman* or palestin* or pakistan* or qatar* or saudi-arabia* or saudi* or somalia* or sudan* or syria* or tunisia* or united-arab-emirates or emirati* or yemen* or middle-east* or north-africa* or west-bank or gaza* or mena or maghreb* or israel* or arab* or druze) | Expanders - Apply equivalent subjects  Search modes - Boolean/Phrase | 120,643 |
| S4 | (MH "Middle East+") OR (MH "Africa, Northern+") OR (MH "Djibouti") OR (MH "Sudan") OR (MH "Mauritania") OR (MH "Pakistan") OR (MH "Somalia") | Expanders - Apply equivalent subjects  Search modes - Boolean/Phrase | 11,861 |
| S3 | S1 OR S2 | Expanders - Apply equivalent subjects  Search modes - Boolean/Phrase | 56,818 |
| S2 | (MH "GLBT Persons+")  or (MH "Gender Nonconformity")  or (MH "Transsexualism")  or (MH "Gender Dysphoria")  or (MH "Gender Identity")  or (MH "Disorders of Sex Development+") | Expanders - Apply equivalent subjects  Search modes - Boolean/Phrase | 9,905 |
| S1 | (gender* N2 nonconform*) or (gender* N2 non-conform*) or (trans N2 (female* or male* or man or men or women or woman or boy* or girl* )) or (trans N2 (population* or patient* or participant* or subject* or adolescent* or teen* or child* or individual* or people or person* or youth*)) or agender* or bicurious or bigender* or bisexual* or cross-sex or crossgender or DSD or gay or gays or gender-change or gender-crossing or gender-dysphori* or gender-fluid* or gender-identit* or gender-incongruen* or gender-minorit* or gender-neutral or gender-queer or gender-transition* or gender-varian* or genderless or genderqueer* or GLB or GLBQ or GLBs or GLBT or GLBTQ or heteroflexible or homosexual* or intersex* or lesbian* or lesbigay* or LGB or LGBQ or LGBS or LGBT* or men-who-have-sex-with-men or mostly-heterosexual or MSM or MSMW or nonbinary or non-binary or nonheterosexual* or non-heterosexual* or queer or queers or same-gender-loving or same-sex-couple* or same-sex-relations* or same-sex-attract* or sexual-identit* or sexual-minorit* or sexual-orientation* or sexual-preference* or SGM or third-gender* or transboy* or transex* or transfeminine or transgender* or transgirl* or transm#n or transmasculine or transsex* or trans-sex* or trans-spectrum or transwom#n or two-spirit* or women-loving-women or women-who-have-sex-with-women or WSW or WSWM | Expanders - Apply equivalent subjects  Search modes - Boolean/Phrase | 54,817 |

| Link to rerun the CINAHL search |
| --- |
| http://search.ebscohost.com/login.aspx?direct=true&db=ccm&bquery=(((gender*+N2+nonconform*)+OR+(gender*+N2+non-conform*)+OR+(trans+N2+(female*+OR+male*+OR+man+OR+men+OR+women+OR+woman+OR+boy*+OR+girl*))+OR+(trans+N2+(population*+OR+patient*+OR+participant*+OR+subject*+OR+adolescent*+OR+teen*+OR+child*+OR+individual*+OR+people+OR+person*+OR+youth*))+OR+agender*+OR+bicurious+OR+bigender*+OR+bisexual*+OR+cross-sex+OR+crossgender+OR+DSD+OR+gay+OR+gays+OR+gender-change+OR+gender-crossing+OR+gender-dysphori*+OR+gender-fluid*+OR+gender-identit*+OR+gender-incongruen*+OR+gender-minorit*+OR+gender-neutral+OR+gender-queer+OR+gender-transition*+OR+gender-varian*+OR+genderless+OR+genderqueer*+OR+GLB+OR+GLBQ+OR+GLBs+OR+GLBT+OR+GLBTQ+OR+heteroflexible+OR+homosexual*+OR+intersex*+OR+lesbian*+OR+lesbigay*+OR+LGB+OR+LGBQ+OR+LGBS+OR+LGBT*+OR+men-who-have-sex-with-men+OR+mostly-heterosexual+OR+MSM+OR+MSMW+OR+nonbinary+OR+non-binary+OR+nonheterosexual*+OR+non-heterosexual*+OR+queer+OR+queers+OR+same-gender-loving+OR+same-sex-couple*+OR+same-sex-relations*+OR+same-sex-attract*+OR+sexual-identit*+OR+sexual-minorit*+OR+sexual-orientation*+OR+sexual-preference*+OR+SGM+OR+third-gender*+OR+transboy*+OR+transex*+OR+transfeminine+OR+transgender*+OR+transgirl*+OR+transm%23n+OR+transmasculine+OR+transsex*+OR+trans-sex*+OR+trans-spectrum+OR+transwom%23n+OR+two-spirit*+OR+women-loving-women+OR+women-who-have-sex-with-women+OR+WSW+OR+WSWM)+OR+((MH+%26quot%3bGLBT+Persons%2b%26quot%3b)+OR+(MH+%26quot%3bGender+Nonconformity%26quot%3b)+OR+(MH+%26quot%3bTranssexualism%26quot%3b)+OR+(MH+%26quot%3bGender+Dysphoria%26quot%3b)+OR+(MH+%26quot%3bGender+Identity%26quot%3b)+OR+(MH+%26quot%3bDisorders+of+Sex+Development%2b%26quot%3b)))+AND+(((MH+%26quot%3bMiddle+East%2b%26quot%3b)%26%23160%3bOR+(MH+%26quot%3bAfrica%2c+Northern%2b%26quot%3b)+OR+(MH+%26quot%3bDjibouti%26quot%3b)+OR+(MH+%26quot%3bSudan%26quot%3b)+OR+(MH+%26quot%3bMauritania%26quot%3b)+OR+(MH+%26quot%3bPakistan%26quot%3b)+OR+(MH+%26quot%3bSomalia%26quot%3b))+OR+((afghan*+OR+algeria*+OR+bahrain*+OR+djibouti*+OR+egypt*+OR+iran*+OR+iraq*+OR+jordan*+OR+kuwait*+OR+lebanon+OR+lebanese+OR+libya*+OR+mauritania*+OR+morocco+OR+morroccan*+OR+oman*+OR+palestin*+OR+pakistan*+OR+qatar*+OR+saudi-arabia*+OR+saudi*+OR+somalia*+OR+sudan*+OR+syria*+OR+tunisia*+OR+united-arab-emirates+OR+emirati*+OR+yemen*+OR+middle-east*+OR+north-africa*+OR+west-bank+OR+gaza*+OR+mena+OR+maghreb*+OR+israel*+OR+arab*+OR+druze))+OR+((Kabul+OR+Algiers+OR+Manama+OR+Djibouti+OR+Cairo+OR+Tehran+OR+Baghdad+OR+Amman+OR+Kuwait-City+OR+Beirut+OR+Tripoli+OR+Nouakchott+OR+Rabat+OR+Muscat+OR+Ramallah+OR+Jerusalem+OR+Islamabad+OR+Doha+OR+Riyadh+OR+Mogadishu+OR+Khartoum+OR+Damascus+OR+Tunis+OR+Abu-Dhabi+OR+Sana%26%2339%3ba+OR+Sanaa)))&cli0=DT1&clv0=200001-202112&type=1&searchMode=Standard&site=ehost-live |

# **PsycINFO**

| # | Searches |
| --- | --- |
| 1 | [PsycINFO search, based on MEDLINE] |
| 2 | (gender* adj2 nonconform*).mp. |
| 3 | (gender* adj2 non-conform*).mp. |
| 4 | (trans adj (female* or male* or man or men or women or woman or boy* or girl*)).mp. |
| 5 | (trans adj (population* or patient* or participant* or subject* or adolescent* or teen* or child* or individual* or people or person* or youth*)).mp. |
| 6 | agender*.mp. |
| 7 | bicurious.mp. |
| 8 | bigender*.mp. |
| 9 | bisexual*.mp. |
| 10 | cross sex.mp. |
| 11 | crossgender.mp. |
| 12 | DSD.mp. |
| 13 | gay.mp. |
| 14 | gays.mp. |
| 15 | gender change.mp. |
| 16 | gender crossing.mp. |
| 17 | gender dysphori*.mp. |
| 18 | gender fluid*.mp. |
| 19 | gender identit*.mp. |
| 20 | gender incongruen*.mp. |
| 21 | gender minorit*.mp. |
| 22 | gender neutral.mp. |
| 23 | gender queer.mp. |
| 24 | gender transition*.mp. |
| 25 | gender varian*.mp. |
| 26 | genderless.mp. |
| 27 | genderqueer*.mp. |
| 28 | GLB.mp. |
| 29 | GLBQ.mp. |
| 30 | GLBs.mp. |
| 31 | GLBT.mp. |
| 32 | GLBTQ.mp. |
| 33 | heteroflexible.mp. |
| 34 | homosexual*.mp. |
| 35 | intersex*.mp. |
| 36 | lesbian*.mp. |
| 37 | lesbigay*.mp. |
| 38 | LGB.mp. |
| 39 | LGBQ.mp. |
| 40 | LGBS.mp. |
| 41 | LGBT*.mp. |
| 42 | men who have sex with men.mp. |
| 43 | mostly-heterosexual.mp. |
| 44 | MSM.mp. |
| 45 | MSMW.mp. |
| 46 | nonbinary.mp. |
| 47 | non-binary.mp. |
| 48 | nonheterosexual*.mp. |
| 49 | non-heterosexual*.mp. |
| 50 | queer.mp. |
| 51 | queers.mp. |
| 52 | same gender loving.mp. |
| 53 | same sex couple*.mp. |
| 54 | same sex relations*.mp. |
| 55 | same-sex attract*.mp. |
| 56 | sexual identit*.mp. |
| 57 | sexual minorit*.mp. |
| 58 | sexual orientation*.mp. |
| 59 | sexual preference*.mp. |
| 60 | SGM.mp. |
| 61 | third gender*.mp. |
| 62 | transboy*.mp. |
| 63 | transex*.mp. |
| 64 | transfeminine.mp. |
| 65 | transgender*.mp. |
| 66 | transgirl*.mp. |
| 67 | transm#n.mp. |
| 68 | transmasculine.mp. |
| 69 | transsex*.mp. |
| 70 | trans-sex*.mp. |
| 71 | trans-spectrum.mp. |
| 72 | transwom#n.mp. |
| 73 | two-spirit*.mp. |
| 74 | women loving women.mp. |
| 75 | women who have sex with women.mp. |
| 76 | WSW.mp. |
| 77 | WSWM.mp. |
| 78 | or/2-77 |
| 79 | sexual minority groups/ |
| 80 | same sex couples/ |
| 81 | same sex marriage/ |
| 82 | exp intersex conditions/ |
| 83 | exp gender identity/ |
| 84 | gender dysphoria/ |
| 85 | or/79-84 |
| 86 | (or/78) or 85 |
| 87 | [MENA region] |
| 88 | arabs/ |
| 89 | (afghan* or algeria* or bahrain* or djibouti* or egypt* or iran* or iraq* or jordan* or kuwait* or lebanon or lebanese or libya* or mauritania* or morocco or morroccan* or oman* or palestin* or pakistan* or qatar* or saudi arabia* or saudi* or somalia* or sudan* or syria* or tunisia* or united arab emirates or emirati* or yemen* or middle east* or north africa* or west bank or gaza* or mena or maghreb* or israel* or arab* or druzez).mp. |
| 90 | (Kabul or Algiers or Manama or Djibouti or Cairo or Tehran or Baghdad or Amman or Kuwait City or Beirut or Tripoli or Nouakchott or Rabat or Muscat or Ramallah or Jerusalem or Islamabad or Doha or Riyadh or Mogadishu or Khartoum or Damascus or Tunis or Abu Dhabi or Sana'a or Sanaa).mp. |
| 91 | or/88-90 |
| 92 | [summation of LGBT and MENA] |
| 93 | 86 and 91 |
| 94 | limit 93 to yr="2000 -Current" |
| 95 | limit 94 to ("0400 dissertation abstract" or conference proceedings) |
| 96 | 94 not 95 |

The Ovid PsycINFO search can be rerun at <http://access.ovid.com/demo/ovidsptools/launcher/dlp_launcher.html>.

# **Embase**

| # | Searches |
| --- | --- |
| 1 | [Dec 19 Embase version based on MEDLINE version] |
| 2 | (gender* adj2 nonconform*).mp. |
| 3 | (gender* adj2 non-conform*).mp. |
| 4 | (trans adj (female* or male* or man or men or women or woman or boy* or girl*)).mp. |
| 5 | (trans adj (population* or patient* or participant* or subject* or adolescent* or teen* or child* or individual* or people or person* or youth*)).mp. |
| 6 | agender*.mp. |
| 7 | bicurious.mp. |
| 8 | bigender*.mp. |
| 9 | bisexual*.mp. |
| 10 | cross sex.mp. |
| 11 | crossgender.mp. |
| 12 | DSD.mp. |
| 13 | gay.mp. |
| 14 | gays.mp. |
| 15 | gender change.mp. |
| 16 | gender crossing.mp. |
| 17 | gender dysphori*.mp. |
| 18 | gender fluid*.mp. |
| 19 | gender identit*.mp. |
| 20 | gender incongruen*.mp. |
| 21 | gender minorit*.mp. |
| 22 | gender neutral.mp. |
| 23 | gender queer.mp. |
| 24 | gender transition*.mp. |
| 25 | gender varian*.mp. |
| 26 | genderless.mp. |
| 27 | genderqueer*.mp. |
| 28 | GLB.mp. |
| 29 | GLBQ.mp. |
| 30 | GLBs.mp. |
| 31 | GLBT.mp. |
| 32 | GLBTQ.mp. |
| 33 | heteroflexible.mp. |
| 34 | homosexual*.mp. |
| 35 | intersex*.mp. |
| 36 | lesbian*.mp. |
| 37 | lesbigay*.mp. |
| 38 | LGB.mp. |
| 39 | LGBQ.mp. |
| 40 | LGBS.mp. |
| 41 | LGBT*.mp. |
| 42 | men who have sex with men.mp. |
| 43 | mostly-heterosexual.mp. |
| 44 | MSM.mp. |
| 45 | MSMW.mp. |
| 46 | nonbinary.mp. |
| 47 | non-binary.mp. |
| 48 | nonheterosexual*.mp. |
| 49 | non-heterosexual*.mp. |
| 50 | queer.mp. |
| 51 | queers.mp. |
| 52 | same gender loving.mp. |
| 53 | same sex couple*.mp. |
| 54 | same sex relations*.mp. |
| 55 | same-sex attract*.mp. |
| 56 | sexual identit*.mp. |
| 57 | sexual minorit*.mp. |
| 58 | sexual orientation*.mp. |
| 59 | sexual preference*.mp. |
| 60 | SGM.mp. |
| 61 | third gender*.mp. |
| 62 | transboy*.mp. |
| 63 | transex*.mp. |
| 64 | transfeminine.mp. |
| 65 | transgender*.mp. |
| 66 | transgirl*.mp. |
| 67 | transm#n.mp. |
| 68 | transmasculine.mp. |
| 69 | transsex*.mp. |
| 70 | trans-sex*.mp. |
| 71 | trans-spectrum.mp. |
| 72 | transwom#n.mp. |
| 73 | two-spirit*.mp. |
| 74 | women loving women.mp. |
| 75 | women who have sex with women.mp. |
| 76 | WSW.mp. |
| 77 | WSWM.mp. |
| 78 | or/2-77 |
| 79 | exp "sexual and gender minority"/ |
| 80 | bisexuality/ |
| 81 | exp gender dysphoria/ |
| 82 | exp homosexuality/ |
| 83 | exp gender identity/ |
| 84 | exp disorder of sex development/ |
| 85 | or/79-84 |
| 86 | 78 or 85 |
| 87 | [MENA region] |
| 88 | exp middle east/ or exp north africa/ or afghanistan/ or djibouti/ or exp pakistan/ or exp somalia/ or sudan/ or south sudan/ |
| 89 | (afghan* or algeria* or bahrain* or djibouti* or egypt* or iran* or iraq* or jordan* or kuwait* or lebanon or lebanese or libya* or mauritania* or morocco or morroccan* or oman* or palestin* or pakistan* or qatar* or saudi arabia* or saudi* or somalia* or sudan* or syria* or tunisia* or united arab emirates or emirati* or yemen* or middle east* or north africa* or west bank or gaza* or mena or maghreb* or israel* or arab* or druze).mp. |
| 90 | (Kabul or Algiers or Manama or Djibouti or Cairo or Tehran or Baghdad or Amman or Kuwait City or Beirut or Tripoli or Nouakchott or Rabat or Muscat or Ramallah or Jerusalem or Islamabad or Doha or Riyadh or Mogadishu or Khartoum or Damascus or Tunis or Abu Dhabi or Sana'a or Sanaa).mp. |
| 91 | exp arabs/ or druze/ or "amhara (people)"/ |
| 92 | exp north african/ |
| 93 | afghan/ |
| 94 | mauritanian/ |
| 95 | pakistani/ |
| 96 | "somali (citizen)"/ |
| 97 | bahraini/ or emirati/ or "iranian (citizen)"/ or iraqi/ or israeli/ or jordanian/ or kuwaiti/ or lebanese/ or omani/ or palestinian/ or qatari/ or saudi/ or syrian/ or yemeni/ |
| 98 | or/88-97 |
| 99 | [summation of LGBT and MENA] |
| 100 | 86 and 98 |
| 101 | limit 100 to yr="2000 - 2020" |
| 102 | limit 101 to (conference abstracts or conference abstract status or (conference abstract or conference paper or "conference review") or conference proceeding) |
| 103 | 101 not 102 |

The Ovid Embase search can be rerun at <http://access.ovid.com/demo/ovidsptools/launcher/dlp_launcher.html>.
