# Supplementary material for: Sexual and gender minority health in the Middle East and North Africa Region: A scoping review
Source: Int J Nurs Stud Adv. 2022 Jun 27;4:100085. doi: 10.1016/j.ijnsa.2022.100085 (PMC11080540; doi:10.1016/j.ijnsa.2022.100085)
Supplement: Supplementary file 2 [file mmc2.docx]

**Articles excluded because of language:**

1. Authors: Alavi, K., Jalali, A. H., & Eftekhar, M.

Title: Sexual orientation in patients with gender identity disorder

Journal and Year: Iranian Journal of Psychiatry and Clinical Psychology;20(1):43-49; 2014

1. Authors: Jalali, A. H., Alavi, K., Pakdel, S., Ahmadzadeh Asl, M., & Eftekhar, M.

Title: Knowledge and attitudes towards AIDS in patients with gender identity disorder presenting to Tehran Psychiatric Institute

Journal and Year: Iranian Journal of Psychiatry and Clinical Psychology;17(3):256-26; 2011

1. Authors: Vasegh Rahimparvar, F., Mousavi, M. S., Rayisi, F., Khodabandeh, F., & Bahrani, N.

Title: Comparison of quality of life in gender identity disorders after sex reassignment surgery with normal women in Tehran, Iran, 2012

Journal and Year: The Iranian Journal of Obstetrics, Gynecology and Infertility;16 (74):10-19; 2013.

1. Authors: Somaye Rahimi, S., Kalantari, M., Abedi, M. R., & Modares, S. M.

Title: The role of early maladaptive schemas and difficulties in emotion regulation in the prediction of Gender Dysphoria in Transsexual men

Journal and Year: Journal of Psychology & Psychiatry;6(3):144-155; 2019

1. Authors: Rahimi Ahmadabadi, S., Kalantari, M., Abedi, M., & Modarres Gharavi, S. M.

Title: Investigating parent-child relationship in predicting gender dysphoria in transsexual women and men

Journal and Year: Journal of Psychology;24(2):200-214; 2020

1. Authors: Klonover, E., Soskolne, V., & Kulik, L.

Title: Risky sexual behavior among gay men in Israel

Journal and Year: Megamot;50(2):153-189; 2016

**Articles excluded because of no full text:**

1. Authors and title:

پورکاظم محمدفريدني, فرزانه; نوگوراني, روناک عشقي

اثربخشي درمان مبتني بر پذيرش و تعهد برافزايش بهزيستي روان شناختي افراد ترا جنسي

Journal and Year: Shenakht Journal of Psychology & Psychiatry 2018;5(1):29-41; 2018

1. Authors: Abidi, M. A. & Ullah, H.

Title: Gender identity disorder

Journal and Year: Journal of the College of Physicians and Surgeons Pakistan;11(4):255-256; 2001

1. Authors: Abidi, A. & Ullah, H.

Title: Gender identity disorder

Journal and Year: Medical Forum Monthly;11(9):23-24; 2000
